# Pt‐Mediated Co‐Adsorption of Hydroxyl and Nitrogen‐Heterocyclic Species Over Cu/CuO Heterostructure Enhances Coupled Azotriazole Electrosynthesis and Dual‐Electrode Hydrogen Production

**DOI:** 10.1002/anie.3766558

**Published:** 2026-08-23

**Authors:** Jiachen Li, Hanggu Su, Yuqiang Ma, Meng Li, Ruhao Zhang, Cihang Wang, Zihang Zhao, Jun Hu, Xiaogang Mu, Xuanjun Wang, Haixia Ma, Zhengxiao Guo

**Affiliations:** ^1^ Xi'an Key Laboratory of Special Energetic Materials School of Chemical Engineering Northwest University Xi'an China; ^2^ Department of Chemistry The University of Hong Kong Hong Kong China; ^3^ Zhijian Laboratory PLA Rocket Force University of Engineering Xi'an China

**Keywords:** co‐adsorption, electrochemical N─N coupling, energy‐saving electrolysis, *N*‐heterocyclic azo compounds

## Abstract

Electrocatalytic N─N oxidative coupling (OC) offers an eco‐friendly approach to produce value‐added azo materials, but suffers from sluggish kinetics and limited Faradaic efficiency (FE). The key fundamental issue is unfavorable competitive adsorption of hydroxyl groups and *N*‐heterocyclic species. Here, a Pt‐immobilized Cu/CuO heterostructure is designed to create energy‐favorable N─N OC of *N*‐heterocyclic diaminotriazole (DATOC). *Operando* spectroscopy along with theoretical calculations reveal that the immobilization of Pt nanoclusters (NCs) in Cu/CuO mediates adsorption capacities for both *N*‐heterocyclic substrates/intermediates and OH^−^ species on Cu sites of Cu/CuO, collaboratively promoting DATOC kinetics. While the N─N intermediates are effectively adsorbed on Pt─Cu sites via metal–N coordination. Consequently, the Pt@Cu/CuO catalyst only requires 0.832 and 0.904 V_RHE_ to achieve 10 and 100 mA cm^−2^, respectively, with a high FE of 95%. Due to the low‐potential DATOC, the adsorbed hydrogen atom from N─H cleavage of aminotriazole undergoes counterintuitive self‐coupling to H_2_. As a result, the dual‐electrode H_2_ production requires an electricity input of only 1.15 kWh per m^3^ of H_2_. The exceptional catalytic durability of the coupled system is also demonstrated in a flow electrolyzer for 600 h@500 mA cm^−2^. This work provides a new perspective for highly efficient green azo electrosynthesis and low‐energy‐consumption H_2_ production.

## Graphical abstract

Pt@Cu/CuO heterostructures were designed for electrochemical N─N coupling of 3,5‐diamino‐1*H*‐1,2,4‐triazole. Mechanism investigation revealed that the Pt sites mediated co‐adsorption of substrates and hydroxyl on Cu/CuO sites. The elaborate Pt@Cu/CuO achieved an ultralow working potential of 0.832 V_RHE_ with a high FE of 95%. A long‐term stability of 600 h was achieved to reach 500 mA cm^−2^, demonstrating a potential for future application.

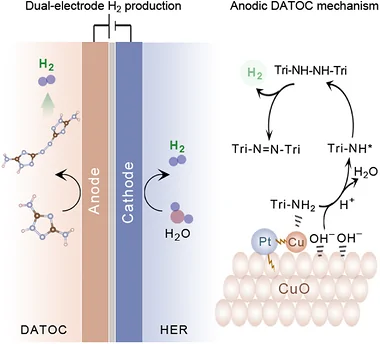

## 1 Introduction

*N*‐heterocyclic azo compounds are characterized by *N*‐functionalized symmetrical or distinct rings with azo groups, and are critical chemicals for applications in bio‐pharmaceuticals, energy materials, agrochemicals, dyes, and textiles [1, 2, 3]. For example, the electron‐rich nitrogen heterocycle could readily accept or donate a proton and establish diverse weak interactions (hydrogen bonding formation, π‐stacking interactions, van der Waals forces) of nitrogen compounds; these special features increase the importance of medicinal chemistry [4]. In addition, typical nitrogen‐rich heterocycles possess a number of inherently energetic N─N/C─N bonds with electronic delocalization feature endows high density and high heat of formation in the field of energetic materials [5]. One of the basic synthetic strategies for nitrogen heterocycle‐based azo compounds is chemical oxidation of corresponding amide starting materials. However, such a process usually involves hazardous heavy metal chemical oxidants (manganese, lead, mercury, etc.), elevated temperature, and an extra separation process of by‐products originated from the chemical oxidants [6, 7]. For example, the synthesis of 5,5'‐diamino‐3,3'‐azo‐1*H*‐1,2,4‐triazole (DAAT) requires ice‐bath treatment of a stoichiometric amount of KMnO_4_, MnO_2_ by‐product separation, and high‐temperature reaction (100°C for 3 h) to realize azo‐bridging (Figure 1a) [8]. Thus, exploration of eco‐friendly synthetic protocols for efficient self‐coupling of nitrogen heterocycle‐based amides is of fundamental and practical significance.

**FIGURE 1:**
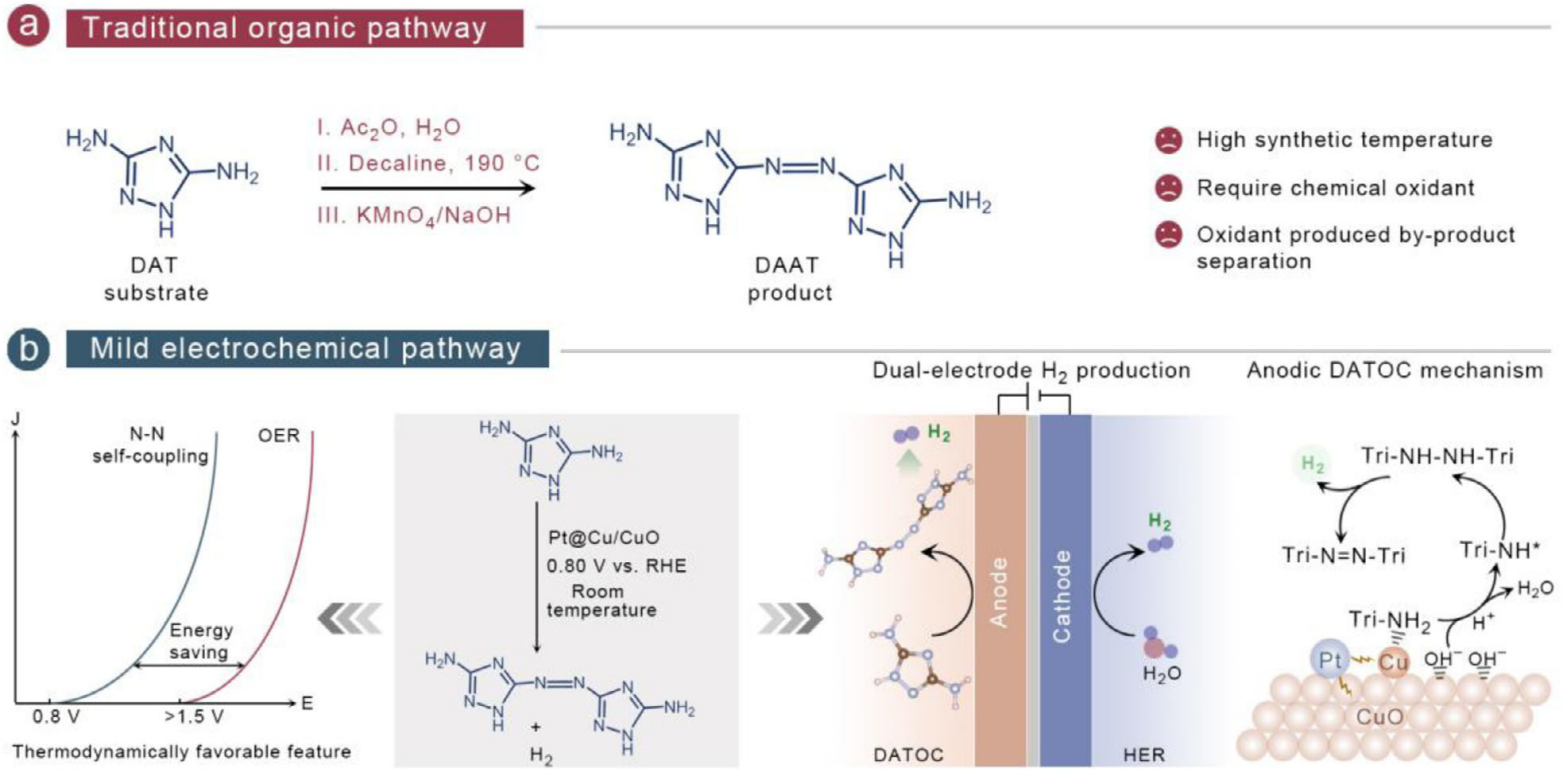
Synthetic strategy of azotriazole compounds. (a) Traditional three‐step pathway with inherent disadvantages. (b) Green electrochemical N─N coupling pathway with a thermodynamically favorable feature, and the proposed mechanism of co‐adsorption of *OH and *NX on Pt@Cu/CuO interfaces.

Organic electrochemistry offers many advantages over traditional synthetic strategies, including environmentally benign, exogenous‐oxidant‐free, and facile reaction conditions [9, 10]. In recent years, there have been various reports on oxidative cross‐coupling of C─C, C─X (heteroatom), and X–Y (heteroatom) to construct new chemical bonds for value‐added chemicals [10, 11]. However, concise electrochemical methods for N─H/N─H oxidative coupling (OC), especially for azo bond formation, are still lacking [9, 12, 13, 14]. Moreover, most electrosynthesis processes only utilize half of the reaction (either anodic or cathodic) for a targeted chemical, while the other half is usually a sacrificial process, such as cathodic hydrogen production. Hence, efficient utilization of the counter reaction can promote the overall efficiency and value of electrosynthesis [15, 16]. Particularly, coupled electrolysis can produce desired products at both electrodes. The energy consumption may be further reduced by thermodynamically favorable oxidation of small organic molecules (alcohols, aldehydes, etc.) [17, 18, 19]. The first example of such coupled electrolysis for the coproduction of value‐added azo compounds and hydrogen was based on thermodynamically favorable N─N OC (0.5 V_RHE_ vs. oxygen evolution reaction (OER), 1.23 V_RHE_) and cathodic HER [20, 21]. Following this, the pairing of the N─N self‐coupling with HER has drawn wide interest for the design of *N*‐heterocyclic substrates and electrocatalysts [22, 23, 24, 25].

Actually, a central challenge, particularly in electrocatalysis, is achieving co‐adsorption for different species to control multiple‐step reactions. Compared with the extensive investigation of low‐potential aldehyde oxidation, the N─N coupling for *N*‐heterocyclic azo compounds is inadequately understood due to the complexity of multiple hydroxy/organics‐coupled electron transfer reactions. The widely recognized mechanism of N─N coupling to an azo product involves stepwise dehydrogenation of –NH_2_ species in *N*‐heterocyclic amide, triggered by the hydroxide radical to form diverse nitrogen intermediates (*NH_2_, *NH, N*, *NH─NH, *N─NH and *N═N) [22, 26]. Such nucleophilic electrooxidation undergoes a direct oxidation pathway, particularly with competing adsorptions of the highly active *OH and the nitrogen intermediates [27]. Consequently, it is difficult to ensure the activity and selectivity of N─N coupling due to the relatively low migration energy and small ionic radius of OH^−^, which is kinetically favored over an *N*‐heterocyclic amide substrate [28]. This will block the active sites and limit amide substrate access. In addition, the excessive OH^−^ adsorption increases the proportion of the oxygen evolution side reaction, which reduces N─N coupling selectivity [29]. In this regard, the concept of co‐adsorption of *OH and nitrogen intermediates (*NX) plays a crucial role for highly efficient N─N coupling, which depends on the rational construction of collaborative multiple active sites to ensure energetically favorable co‐adsorption configurations.

Rational design of heterostructures with a clear strategy for multiple active site engineering enables fine‐tuning of the adsorption behavior of *OH and *NX organics [30]. Such a strategy can well resolve the restricted exposure of active sites of mainstream Cu‐based catalysts for the oxidation of small organic molecules [20, 31]. In addition, the incorporation of a secondary component (e.g., Pt) can potentially address the issue of sluggish reaction kinetics by mediating the binding energy scaling constraint of the reactants and *OH [32]. Based on the above analysis, we designed heterogeneous Pt@Cu/CuO nanowires (NWs) supported over a copper foam (CF) for electrochemical N─N OC of *N*‐heterocyclic 3,5‐diamino‐1*H*‐1,2,4‐triazole (DAT) (Note: the process is subsequently defined as “DATOC”) with dual‐electrode hydrogen production. The electrodeposition of Pt led to in situ reconstruction of CuO to a partially reduced valence‐gradient Cu/CuO interfacial structure. *Operando* spectroscopy and theoretical calculations reveal that Pt mediates the co‐adsorption of hydroxyl and DAT molecules on CuO and Cu nanocluster (NC) sites, respectively, whereas the triazole intermediate species (*NX═NH─NH*, NH─N*, and N═N*) are preferentially adsorbed on Pt─Cu NC sites, with a much reduced energy barrier for the rate‐determining step (NH─NH*→NH─N*) (Figure 1b).

There remains a practical challenge of market‐size mismatch between oxidative and hydrogenated products and a fundamental challenge of kinetic/mass‐transfer mismatch between anodic and cathodic reactions, leading to relatively high onset potential (> 1 V) and unsatisfactory current density [31, 33]. Counterintuitive anodic HER, triggered by low‐potential oxidation of small organic molecules (aldehydes [31, 34, 35, 36, 37] and *N*‐heterocyclic amides [26]), holds the potential for dual‐electrode hydrogen production and chemical upgrading. The desorption of H* from the amino group in DAT undergoes self‐coupling to H_2_ via the Tafel pathway. Therefore, the increased electron/hydrogen atom utilization makes such novel bipolar electrolysis deliver a high energy conversion efficiency and ultralow energy consumption, while reducing the mismatch of the two electrodes.

Building on a previous report on electrochemical N─N coupling dehydrogenation on a CuO electrocatalyst with simultaneous production of H_2_ at the anode [38], this work addresses the challenges of the sluggish kinetics and limited Faradaic efficiency (FE) of the N─N coupling process. Fundamentally, the competitive adsorption mechanisms of *N*‐heterocyclic intermediates and hydroxyl were probed by experimental design, operando characterizations, and DFT calculations. Practically, multiple active sites (Cu NC, CuO, and Pt NC) in Pt@Cu/CuO heterostructures were comparatively assessed, leading to the discovery of a Pt@Cu/CuO catalyst that only required as low as 0.832 and 0.904 V_RHE_ to achieve 10 and 100 mA cm^−2^ with a high FE of 95% for DATOC. A flow electrolyzer‐driven DATOC operated stably over 600 h to reach an industrial‐level current density of 500 mA cm^−2^, demonstrating its great potential for commercial deployment.

## 2 Results and Discussion

### 2.1 Structural Characterizations of Pt@Cu/CuO Electrocatalysts

The in situ fabrication of Pt‐loaded Cu/CuO NWs over a CF support is shown in Figure 2a. Typically, Cu(OH)_2_ NWs supported on CF (Cu(OH)_2_@CF) were prepared by in situ wet‐chemical oxidation (Figure S1). Subsequently, the obtained Cu(OH)_2_@CF was then annealed under an argon atmosphere to form CuO@CF NWs. Scanning electron microscopy (SEM) shows uniform dispersion of CuO NWs on the CF support (Figures 2b and S2a). Subsequently, the CuO@CF was immersed in a 1.0 M KOH electrolyte with a specified concentration of Pt ion solution. The Pt electrodeposition was conducted by a cyclic voltammetry (CV) strategy (detailed method is shown in the Supporting Information). SEM image of Pt@Cu/CuO shows the coarse surface and preserved NW morphology as Cu/CuO, and the SEM image at a high magnification shows dense Pt NCs dispersed uniformly on the Cu/CuO NWs (Figures 2c and S2b). Transmission electron microscopy (TEM) of Pt@Cu/CuO further identified the NW morphology with some nanopores along the NW, which may be generated by the bubbling and migration of oxygen species and the in situ partial reduction of CuO to metallic Cu during the electrodeposition of Pt NCs (Figure 2d) [39]. The special nanoporous structure of Pt@Cu/CuO promotes the access of electrolyte and improves the available active site density for highly efficient DATOC. High‐resolution TEM (HRTEM) of Pt@Cu/CuO shows an evident hetero‐nanostructure at the surface of the NW (Figure 2e). The distinctive lattice spacings of 0.233, 0.216, and 0.256 nm can be assigned to Pt (111), Cu (111), and CuO (002) crystal planes, respectively, indicating the coexistence of the Pt/Cu/CuO interfaces. Elemental distribution maps further confirm the coexistence of Pt, Cu, and O elements throughout the NW (Figure 2f–i). The x‐ray diffraction (XRD) patterns of the prepared catalysts are shown in Figure S3. The diffraction peaks appearing at 16.7°, 34.1°, 39.7°, and 53.3° are assigned to the characteristic lattice planes of (020), (002), (130), and (150) for Cu(OH)_2_, respectively (JCPDS no. 35–0505) [40]. The other three sharp peaks at 43.3°, 50.5°, and 74.1° are indexed to the metallic Cu in CF support. Moreover, the other peaks at 35.5°, 36.6°, 42.6°, and 61.7° of the XRD pattern of CuO@CF are attributable to CuO (JCPDS no. 45–0937 and 78–0428) [21]. After the Pt NC immobilization, a newly emerged broad peak around 40° is assigned to metallic Pt (JCPDS no. 87–0647), and the intensities of the diffraction peaks for CuO are reduced. This is indicative of partial surface reduction of CuO to metallic Cu during the electrodeposition of Pt NCs.

**FIGURE 2:**
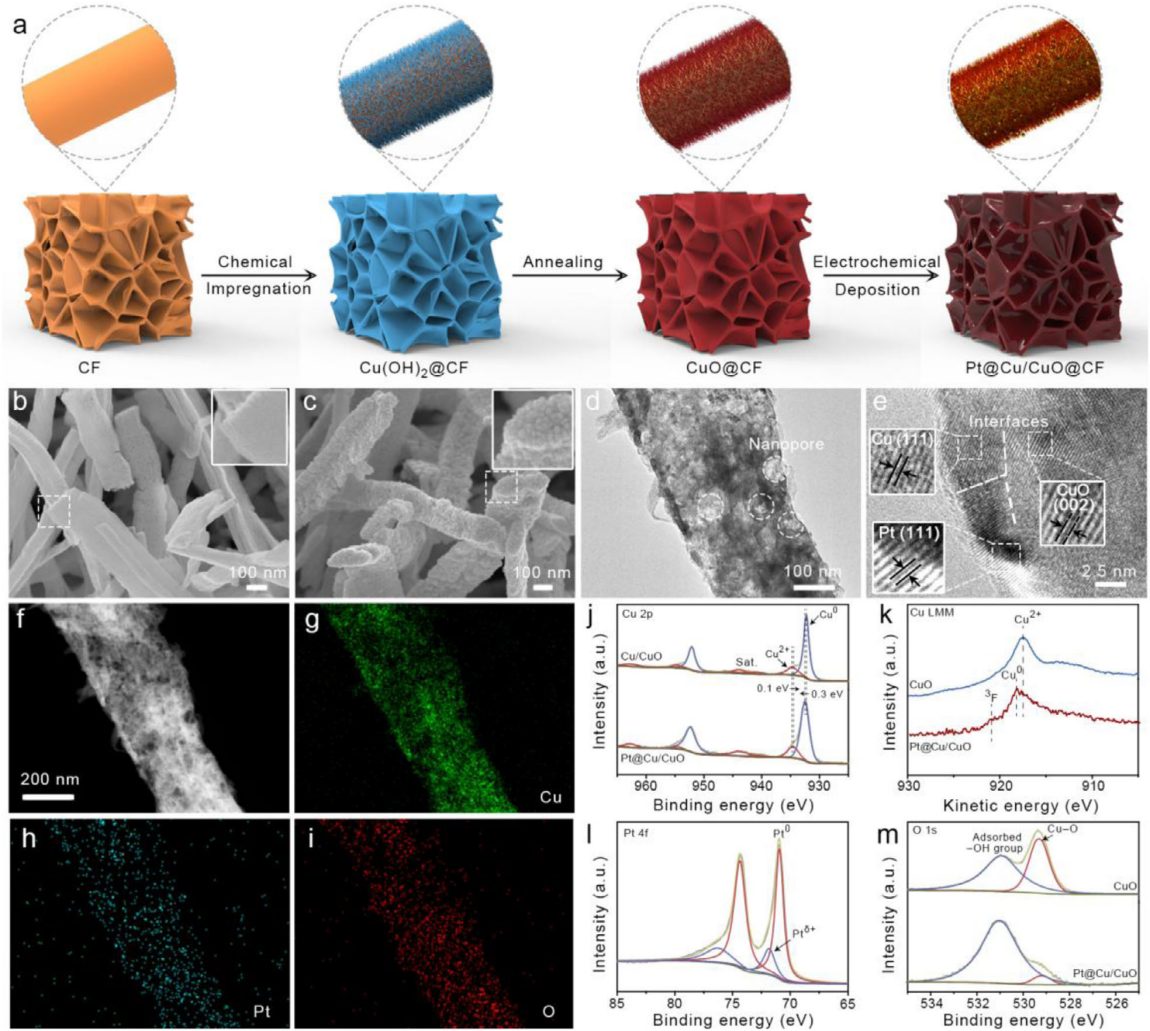
Structural characterizations of Pt@Cu/CuO@CF. (a) Schematic of the Pt@Cu/CuO@CF construction procedure. SEM images of (b) CuO NWs and (c) Pt@Cu/CuO NWs on copper foam support (inset: the magnified SEM image of CuO and Pt@Cu/CuO NWs). (d, e) TEM images of Pt@Cu/CuO NWs. (f) HAADF‐STEM and (g–i) elemental mapping image of Pt@Cu/CuO NWs. (j) High‐resolution XPS spectra of Cu 2p and (k) Cu LMM for CuO and Pt@Cu/CuO. High‐resolution XPS spectra of (l) Pt 4f signal for Pt@Cu/CuO, and (m) O 1s signal for CuO and Pt@Cu/CuO.

X‐ray photoelectron spectroscopy (XPS) was conducted to further reveal the detailed structural information of Pt@Cu/CuO. As shown in Figure 2j, high‐resolution XPS spectra of the Cu 2p signal for Cu/CuO show two dominate peaks at 932.2 and 952.1 eV, which can be assigned to Cu^0^/Cu^+^ species [41]. Two weak peaks at 934.7 and 954.6 eV, along with the fingerprint satellite peaks at 940.9, 943.8, and 962.3 eV, are attributed to Cu^2+^ in the CuO species [42]. The co‐existence of Cu^0^ and CuO induced by the electric field during electrodeposition of Pt NCs was further verified by HRTEM images (Figure S4). After the immobilization of the Pt NCs, a binding energy shift is observed to 0.1 eV shifting of Cu^2+^ from Cu/CuO to Pt@Cu/CuO, and 0.3 eV shifting of Cu^0^/Cu^+^ from Pt@Cu/CuO to Cu/CuO, indicating the electron configuration after Pt NCs loading on Cu/CuO.

In order to further distinguish the Cu° from the Cu^+^ state in the Cu 2p spectra, the Cu Auger L_3_M_45_M_45_ transition state of Cu in Pt@Cu/CuO was then analyzed (Figure 2k). Two final‐state terms of ^1^G and ^3^F corresponding to the Cu Auger excitation spectrum are observed. The most intense ^1^G peak located at 917.5 eV is indicative of Cu^2+^ in CuO [43]. In addition to the Cu^2+^ peak, two new peaks appear at 918.2 eV (^1^G) and 920.6 eV (^3^F) are detected in Pt@Cu/CuO. The distinct ^3^F peak is the fingerprint peak for Cu^0^, further indicating the coexistence of metallic Cu and CuO in Pt@Cu/CuO [44]. Moreover, the doublets of Pt 4f spectra at 70.9 and 74.3 eV are ascribed to Pt^0^ in Pt@Cu/CuO. The other paired peaks located at 71.8 and 76.3 eV are assigned to oxidized Pt due to its inevitable re‐oxidation in air [45]. The O 1s signals also show two main peaks at 529.3 and 531.1 eV, respectively, corresponding to the lattice oxygen in CuO and the adsorbed –OH group [46]. Notably, the increased intensity of the –OH signal and decreased intensity of the Cu─O signal implies the preferential –OH adsorption on the CuO site of Pt@Cu/CuO, so that the Cu─O signal is attenuated. This conclusion is verified further by subsequent theoretical calculations.

### 2.2 Electrochemical N─N Coupling of DATOC

The DATOC electrocatalytic performance of Pt@Cu/CuO was initially optimized by Pt ion concentration, the number of CV cycles during the electrodeposition of Pt NCs, and the DAT concentration in a three‐electrode configuration cell. Figure S5 shows the DATOC activity on Pt@Cu/CuO under various DAT concentrations. Increasing DAT concentration increases the anodic current response. The Pt@Cu/CuO exhibits the highest DATOC current density and the lowest working potential of 0.832 V_RHE_ to reach 10 mA cm^−2^ under 1.0 M KOH with the addition of 0.2 M DAT. Unless otherwise specified, the following electrochemical evaluation was performed under 1.0 M KOH+0.2 M DAT. In addition, we investigated the number of CV cycles and Pt ion concentration toward DATOC activity (Figure S6). After that, the DATOC activity of Pt@Cu/CuO was assessed by linear sweep voltammetry (LSV), and the role of each component in Pt@Cu/CuO was preliminarily discussed via reference samples (Figure 3a). Compared with the reference samples of CF support, CuO, Cu/CuO, and Pt NCs loaded on a carbon cloth support (Pt@CC, Figure S7), the Pt@Cu/CuO heterostructure shows the lowest working potential of only 0.832 and 0.904 V_RHE_ to achieve 10 and 100 mA cm^−2^ for DATOC, respectively. The result verifies the potential synergy among Pt, Cu, and CuO species, which are further elucidated via operando characterizations and theoretical calculations in the following sections. Due to the low‐potential feature of DATOC, the dehydrogenated H* of the DAT substrate may undergo Tafel pathway (H* + H* → H_2_) to form H_2_, instead of the conventional Volmer pathway for low‐value H_2_O (*H + OH^−^ → * + H_2_O + e^−^) [31].

**FIGURE 3:**
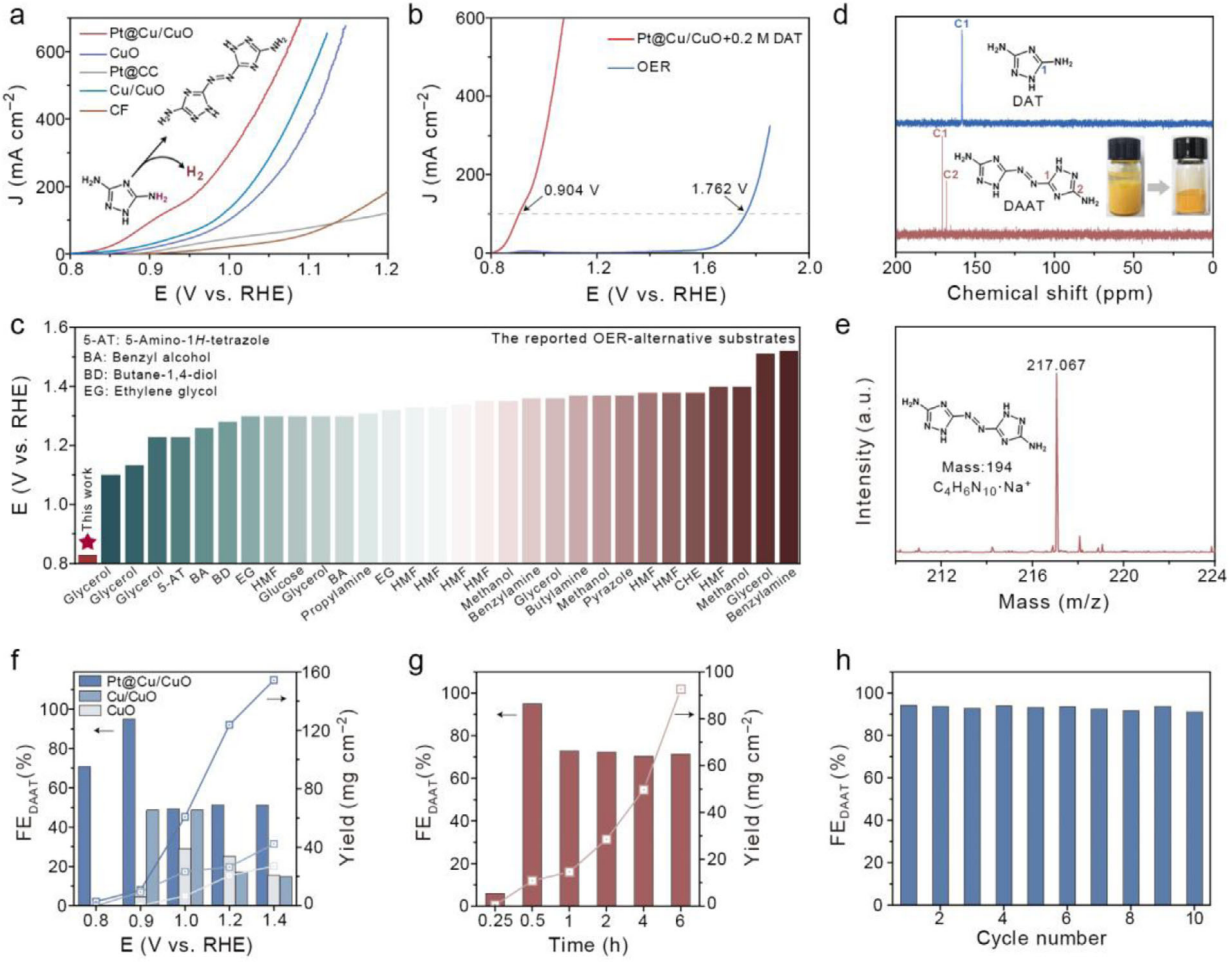
Electrochemical N─N coupling performance. (a) LSV curves of DATOC on Pt@Cu/CuO and reference samples in 1.0 M KOH+0.2 M DAT. (b) LSV curves of DATOC and OER on Pt@Cu/CuO in 1.0 M KOH with or without the addition of 0.2 M DAT. (c) Comparison of working potential of DATOC with reported mainstream organic small molecule oxidation reactions. (d) ^13^C NMR spectra of DAT substrate and DAAT product (inset: Optical photograph of acidified electrolyte and DAAT powder). (e) Mass spectrum of DAAT product. (f) Faradaic efficiency and yield of Pt@Cu/CuO and reference samples under various applied potentials. (g) Electrolysis time optimization at 0.9 V_RHE_ on Pt@Cu/CuO. (h) Recycling performance of Pt@Cu/CuO for DATOC at 0.9 V_RHE_ for 0.5 h.

To prove the thermodynamically favorable N─N coupling of DATOC, the LSV of DATOC and OER on Pt@Cu/CuO was performed in 1.0 M KOH+0.2 M DAT and 1.0 M KOH, respectively (Figure 3b). A voltage of 1.762 V_RHE_ was required to reach 100 mA cm^−2^ for OER, while a marked voltage reduction of 858 mV was observed after the injection of 0.2 M DAT, suggesting the kinetically and thermodynamically favored choice of DATOC, rather than OER. Inspiringly, the applied working potential of DATOC on Pt@Cu/CuO is lower than the majority of mainstream small organic molecule oxidation reactions, demonstrating the high promise of DATOC triggered by Pt@Cu/CuO to broaden the paired electrolysis for diverse applications (Figure 3c and Table S1).

After long‐term electrolysis, the electrolyte is acidized, and the DAAT product precipitates because of the poor solubility of DAAT under low pH. Thus, the DAAT can be readily separated by simple filtration without extra energy‐extensive process such as rotary evaporation and column chromatography. The obtained luminous yellow DAAT powder was characterized by ^13^C nuclear magnetic resonance (NMR) spectroscopy (Figure 3d). Two kinds of chemical shifts at *δ* = 168.3 and 171.0 ppm are attributed to C─NH_2_ and C─N═N, respectively, which are different from the DAT substrate [8]. The formation of DAAT was further verified by mass spectrometry (MS), as shown in Figure 3e. One sharp peak with a mass‐to‐charge ratio of 217.067 is noted, which is indicative of the mass of DAAT and coordinated with exogenous Na^+^. The above discussion indicates the successful synthesis of DAAT via the electrochemical N─N coupling strategy.

Further, the FE and yield of DATOC on Pt@Cu/CuO and reference samples were calculated based on the results from the UV–vis spectrophotometry (Figures 3f and S8). The potential‐relevant FE and yield were first evaluated. As noted in Figures 3g, S9, and S10, Pt@Cu/CuO shows the highest FE of 95.0% at a low potential of 0.9 V_RHE_ after 0.5‐h electrolysis, significantly outperforming those of Cu/CuO (48.8%) and CuO (29.3%). In addition, Pt@Cu/CuO achieves the highest DAAT yield of 154.5 mg cm^−2^ at 1.4 V_RHE_, compared with Cu/CuO (42.4 mg cm^−2^) and CuO (27.2 mg cm^−2^) at the same applied potential. These results suggest that the heterostructure engineering of Cu/CuO and further immobilization of Pt NCs greatly enhance the DATOC efficiency. The conversion rate of DAT, selectivity, and yield of DAAT were quantitatively calculated and exhibited a volcano pattern over the potential ranges of 0.8∼1.4 V_RHE_ (Figure S11). The optimal DAT conversion rate reached 92.03%, with a DAAT yield of 89.64%. Given this, a high selectivity of 97.43% was achieved, indicating the high utilization of the DAT substrate.

Moreover, the Pt@Cu/CuO electrode could be utilized repeatedly and exhibit no evident FE decay within an assessment of 10 cycles upon electrolyte replacement in each cycle, demonstrating the superior DATOC durability of Pt@Cu/CuO (Figure 3h). The morphological and structural characterizations of Pt@Cu/CuO were performed after 2000 cycles of CV tests. As shown in Figure S12, Pt@Cu/CuO still keeps the NW morphology with some aggregation. TEM image exhibits some dark dots over the NWs. HRTEM image confirms the coexistence of Pt NCs, Cu, and CuO. The structure of each component was further verified by XPS. High‐resolution Cu 2p signal of Pt@Cu/CuO shows a decreased amount of Cu^0^ at 532.7 eV compared with the initial state (Figure 2j), indicating that part of the metallic Cu has been oxidized to CuO, which becomes the major component. The Pt 4f spectrum showing two peaks at 71.9 and 74.8 eV is attributed to Pt^0^ species, indicating the stable structure of Pt NCs during DATOC.^[32]^ Long‐term FE tests were then evaluated in an H‐type cell under potentiostatic conditions of 0.9 V. The consecutive electrolysis can be maintained over 200 h with minor fluctuations of FE, indicating highly efficient DATOC over long‐term stable electrolysis (Figure S13).

### 2.3 Origin of Anodic H_2_ Production and Reaction Pathway

The intriguing phenomenon of low‐potential DATOC motivated us to probe further the underlying dehydrogenation mechanism and reaction pathway. Differential electrochemical mass spectrometry (DEMS) was conducted to identify gaseous products during DATOC. A three‐electrode system was employed in the DEMS using Pt@Cu/CuO and Cu/CuO as the working electrode, Pt wire as the counter electrode, and Hg/HgO as the reference electrode, respectively (Figure 4a). The gaseous products were produced during the LSV test and transferred to the detector. The mass signal was detected as a function of the relative time during the LSV test over the potential range of 1.3 ∼ 1.7 V_RHE_ on Pt@Cu/CuO (Figure 4b). Interestingly, the signal at *m/z* = 2 (H_2_) was detected during DATOC, while the signals at *m/z* = 3 (HD) and *m/z* = 4 (D_2_) show no response, indicating that the H atom in H_2_ may be originated from the dehydrogenation of amino groups in the DAT substrate. The H_2_ signal (*m/z* = 2) is less prominent on Cu/CuO than on Pt@Cu/CuO, suggesting that the introduction of Pt NCs is inclined to promote kinetic of DAT dehydrogenation and Tafel recombination of H* to hydrogen molecules (Figure S14). In addition, the flattened signals of *m/z* = 32 (O_2_) and *m/z* = 44 (CO_2_) indicate that the side reactions of OER and over‐oxidation of DAT are not involved in DATOC. For comparison, the DEMS of OER in 1.0 M KOH without injection of DAT was measured over the potential range of 1.0 ∼ 2.0 V_RHE_ (Figure 4c). The onset potential of OER is detected at 1.70 V_RHE_, which is much higher than that of DATOC on Pt@Cu/CuO, demonstrating the low‐potential DATOC. In order to demonstrate the dual‐electrode hydrogen production, the related products in the counter electrode (cathode) are identified. For the proof of concept, the cathode gaseous products were analysed by DEMS (Figure S15). The signal at *m/z* = 2 (H_2_) was detected, and the signals at *m/z* = 32 (O_2_) and *m/z* = 4 (CO_2_) showed no response, demonstrating cathodic hydrogen production and thus confirming the proposed dual‐electrode H_2_ generation.

**FIGURE 4:**
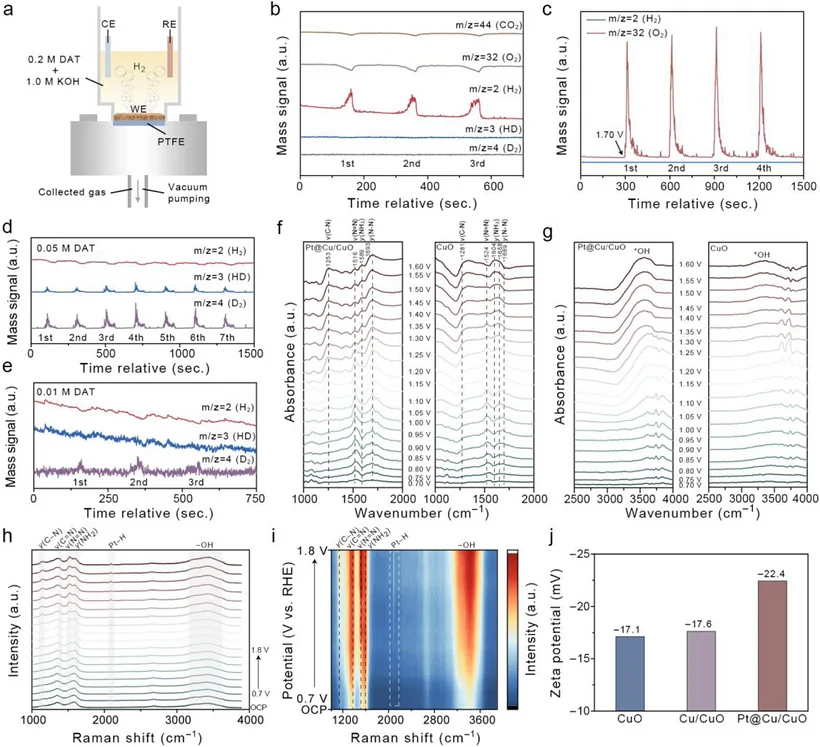
*Operando* characterizations and surface microenvironment exploration during anodic DATOC. (a) Schematic illustration of electrolyzer assembled with DEMS. (b) In situ DEMS spectra of DATOC on Pt@Cu/CuO in 1.0 M KOH+0.2 M DAT. (c) In situ DEMS spectra of OER on Pt@Cu/CuO in 1.0 M KOH. (d, e) In situ DEMS spectra of N─N coupling of deuterated DAT substrate (0.05 M and 0.01 M). (f, g) In situ ATR‐FTIR spectra of DATOC on Pt@Cu/CuO and CuO over the potential range of 0.70 V_RHE_ to 1.60 V_RHE_. (h, i) In situ Raman spectra of DATOC on Pt@Cu/CuO. (j) Zeta potential of Pt@Cu/CuO, Cu/CuO, and CuO in 1.0 M KOH+0.2 M DAT.

The proposed H_2_ production mechanism was further verified via DEMS together with isotope labelling. The DAT was deuterated, and the gaseous products during DATOC were detected. As shown in Figure 4d, the *m/z* = 2 (H_2_) signal is absent, with a signal surge at *m/z* = 4 (D_2_) and a weak signal at *m/z* = 3 (HD). The mass signal at *m/z* = 3 (HD) is absent when the concentration of DAT is reduced, with the signal at *m/z* = 4 (D_2_) still exists (Figure 4e). These results demonstrate that the evolved H_2_ during low‐potential DATOC are originated exclusively from amino dehydrogenation. As a result, the produced H* from amino dehydrogenation undergoes Tafel recombination to form H_2_, instead of the Volmer pathway to release less‐valuable water molecules, leading to the promoted hydrogen/electron economy.

Electrochemical potential‐dependent in situ infrared spectroscopy (IR) of DATOC was then performed on Pt@Cu/CuO to identify the variation of adsorbed intermediates and explore the possible reaction pathway. Control experiments were conducted using CuO as electrocatalysts for DATOC. As shown in Figure 4f, the key spectral feature at ∼1589 cm^−1^ in Pt@Cu/CuO is assigned to N─H stretching of amino groups [47]. The emergence of peaks at ∼1516 and ∼1253 cm^−1^ corresponds to the targeted N═N and C─N bonds, respectively, indicating the successful formation of azo DAAT [47]. In addition, the key ─NH─NH─ intermediates during DATOC were detected at the location of ∼1693 cm^−1^, suggesting that the ─NH─NH─ pathway, rather than the nitrene pathway, dominates the DATOC reaction [48]. Compared with CuO, the peak intensity of ─NH─NH─ increases with the amount of adsorbed –NH_2_ groups, implying that the incorporation of Pt NCs and Cu NCs promotes –NH_2_ dehydrogenation and N─N coupling to ─NH─NH─ intermediates (Figure S16). After the incorporation of Pt NCs, the high intensity of ─NH─NH─ key intermediate was still maintained, while the peak for the azo bond weakened at higher applied potential, indicating that the Pt NCs can stabilize intermediates and promote desorption of DAAT. These observations were further verified by the results of DFT calculations in a later section.

The possible H* intermediates during DATOC were then identified; an absorption band emerged at 2152 cm^−1^, which is assigned to Pt─H stretching vibration in Pt@Cu/CuO (Figure S17) [49]. The Pt─H stretching vibration was intensified progressively upon positive potential shifts, while the Pt─H stretching vibration was not detected in Cu/CuO, indicating that the Pt NCs were the active sites for H* adsorption. Meanwhile, the key adsorbates of N─H and ─NH─NH─ stretching vibrations showed the same tendency as the Pt─H stretching vibration. These results suggested that the competition between N─N coupling and H* recombination was not involved in DATOC. Instead, the H* produced from DAT dehydrogenation were trapped at Pt NCs sites and functioned as “active hydrogen reservoir” for subsequent hydrogen evolution via Tafel recombination. This result was further revealed by in situ Raman spectra, as discussed below. In order to elucidate the role of *OH toward DATOC, the *OH adsorption behavior is revealed by the broad feature at ∼3560 cm^−1^ (Figure 4g) [50]. The intensity of the *OH bands for Pt@Cu/CuO is much higher than those for CuO and Cu/CuO, indicating that the incorporation of Pt NCs enhances *OH adsorption to mediate the dehydrogenation of the –NH_2_ groups.

The adsorption evolution of the *N*‐heterocyclic substrate and hydroxyl was further probed by potential‐resolved surface‐enhanced Raman spectroscopy over the potential range from 0.70 to 1.80 V_RHE_ (Figure 4h,i). The peaks at 1354 and 1578 cm^−1^ are observed at the open‐circuit potential (OCP) stage, which are assigned to triazole ring breathing in DAT molecules [47]. Another broad peak located at 3100∼3700 cm^−1^ is attributed to hydroxyl adsorption, demonstrating the stable co‐adsorption of substrate and hydroxyl on Pt@Cu/CuO. Upon applying a more positive potential, the vibration peaks at 1140, 1383, 1524, and 1600 cm^−1^ are gradually strengthened, which are ascribed to vibrations of γ(C─N), v(C═N), v(N═N), and γ(NH_2_) bonds, respectively [47]. While another peak located at 2093 cm^−1^ is assigned to Pt─H vibration bond, which almost simultaneously appeared as the v(N═N) bond was detected [51, 52]. This result indicates that the Pt NC is the true active site for H* adsorption via N─N coupling dehydrogenation of DAT substrates; the co‐adsorption of N‐related intermediates, hydroxyl species, and H* intermediates is confirmed during DATOR.

Zeta potential reflects the chargeability of electrocatalysts surface and deeply reveals interaction between the catalysts and hydroxyl groups [53]. Therefore, the adsorption behavior of OH^−^ on Pt@Cu/CuO, Cu/CuO, and CuO was characterized by Zeta potential measurement (Figure 4j). Evidently, a more negative potential (–22.4 mV) in an alkaline environment for Pt@Cu/CuO than that of CuO (–17.1 mV) and Cu/CuO (–17.6 mV) manifests that more *OH are enriched in the inner Helmholtz layer of Pt@Cu/CuO and are conducive to the initiation of the DATOC reaction. In addition, the OCP was performed to detect the adsorbed DAT content in the inner Helmholtz layer [54]. With the injection of 0.2 M DAT in 1.0 M KOH, an evident OCP change of Pt@Cu/CuO (Δ*E* = 33.9 mV) was observed compared with CuO (Δ*E* = 24.6 mV) and Cu/CuO (Δ*E* = 25.8 mV), suggesting that more DAT substrates are adsorbed in the inner Helmholtz layer of Pt@Cu/CuO (Figure S18a). This result also indicates that the introduction of Pt NCs on Cu/CuO is beneficial to the adsorption of the DAT substrate dynamically. The adsorption behavior of *OH was further evaluated by CV tests (Figure S18b). A significant oxidation peak is observed at about 0.83 V_RHE_ for Pt@Cu/CuO, which is attributed to the *OH adsorption process (OH^−^ → *OH + e^−^) [55, 56]. The co‐adsorption configuration effect on different levels of Pt loading (controlled by the electrodeposition cycles) in Pt@Cu/CuO was then analyzed by OCP, CV, and Zeta potential measurements. The OCP peak drop of 33.9 mV was observed at 400 cycles (Pt@Cu/CuO), with less pronounced drops recorded for either fewer or greater cycle counts. This result indicated that the adsorption density of DAT molecules within the inner Helmholtz layer increases with the amount of Pt loading. However, more Pt loading amount (>400 cycles) shows a negative effect on DAT adsorption (Figure S19a). CV measurements showed an oxidation peak at about 0.83 V_RHE_ for 400 cycles. Similar to the results in the OCP measurement, increasing Pt loading is conducive to enhancing the *OH adsorption capacity, but insufficient and excessive Pt loadings are not favored for accelerating *OH adsorption (Figure S19b). Zeta potential measurements also showed that the 400 cycles delivered the lowest surface potential, further revealing a higher probability to accumulate OH^−^ species for subsequent DAT oxidation steps (Figure S19c). Based on the above discussion, it is reasonable to infer that the immobilization of Pt NCs and optimization of Pt NC loading amount on Cu/CuO is beneficial to mediating the co‐adsorption of *OH and DAT substrates to achieve highly efficient DATOC.

Based on the above discussion, the key to realize anodic hydrogen production via the Tafel pathway rather than Volmer oxidation lies in the amino group dehydrogenation of DAT at relatively low potentials [31, 57]. To this end, the feature of a thermodynamically favorable electrooxidation reaction and regulation of H* adsorption dynamics following the low‐potential pathway are crucial for anodic hydrogen production. Apart from thermodynamically favorable DATOC (∼0.5 V_RHE_), the regulation of H* adsorption dynamics on Pt@Cu/CuO can lead to hydrogen production at the anode rather than water molecule formation. Typically, the Pt NCs in Pt@Cu/CuO were confirmed for H* adsorption via Pt─H bonding, as evidenced by the detection of Pt─H vibration from situ ATR‐FTIR and Raman spectra. The adsorbed H* from DAT dehydrogenation is preferred to be recombined on Pt sites for hydrogen production. While the reaction from (*H+OH^−^) to (*H+*OH) in the Volmer step was demonstrated to be sluggish on Pt sites, indicating that the incorporation of Pt NCs is beneficial for the Tafel hydrogen production pathway [58].

### 2.4 Theoretical Insight of DATOC Mechanism

In the electrocatalytic process, active site engineering is crucial for accelerating the kinetics of multi‐step molecular oxidation. Moreover, the electronic transport capability plays a fundamental role in influencing the catalytic efficiency and selectivity [59]. In view of this, we constructed and fully relaxed the optimal models of Pt@Cu/CuO and Cu/CuO heterostructures to clarify further the specific roles of the active sites in DATOC at the electronic/atomic scale. Details of the electronic structural calculations are provided in the Supporting Information. The charge distribution difference and Bader charge analysis show that more electron is transferred from Pt NC (0.34 e^–^) and Cu NCs (1.46 e^–^) to CuO in Pt@Cu/CuO than Cu NCs (1.05 e^–^) to CuO in Cu/CuO, indicating that the immobilization of Pt NC increases the electron‐withdrawing of the Cu sites in CuO and Cu NC for substrate and *OH activation, favoring DATOC reaction activity (Figure 5a) [60].

**FIGURE 5:**
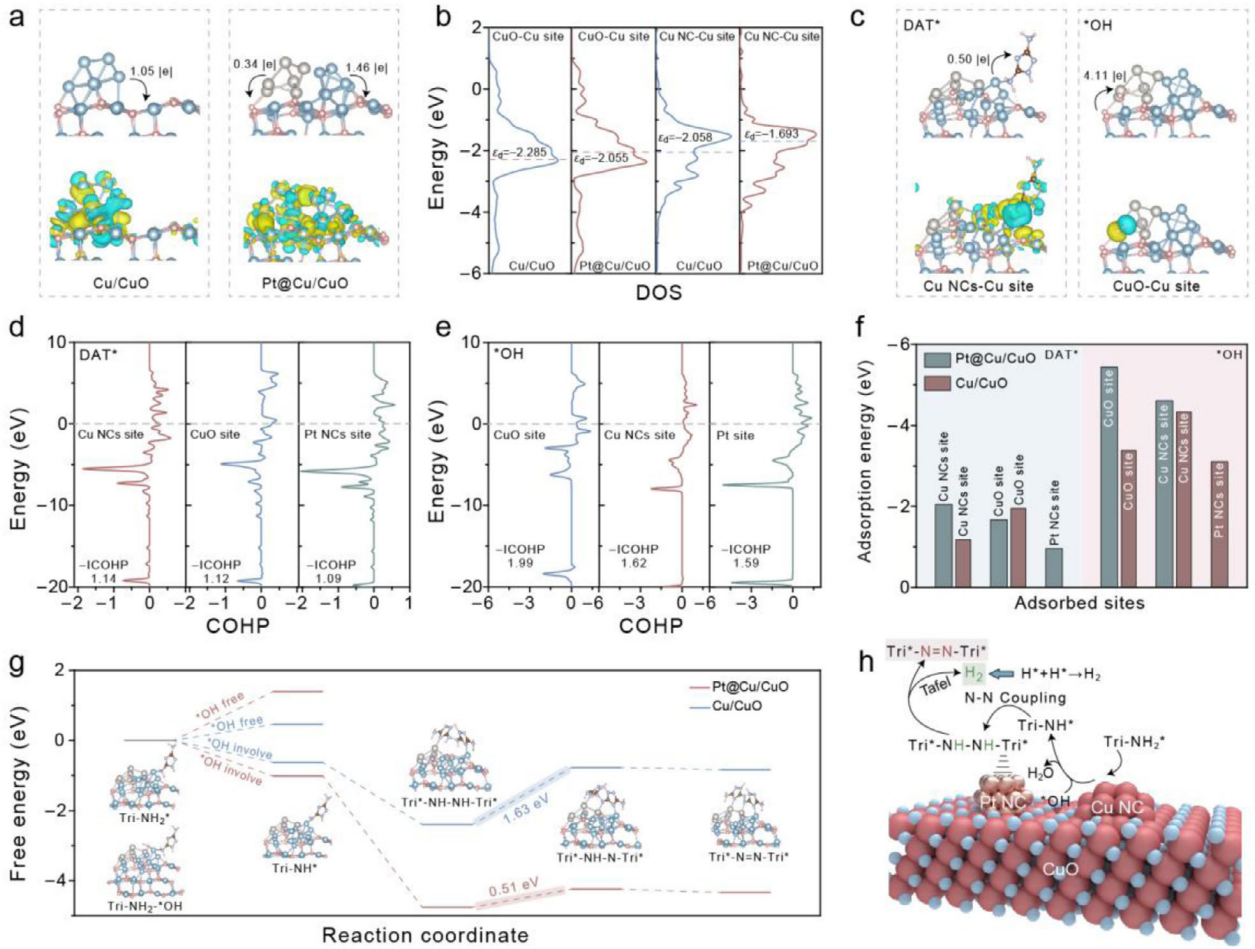
DFT calculations of DATOC on various adsorption sites of Pt@Cu/CuO configurations. (a) Charge density difference and Bader charge analysis on Cu/CuO and Pt@Cu/CuO. The yellow and cyan regions indicate charge accumulation and depletion, respectively. (b) PDOS analysis on CuO and Cu sites of Pt@Cu/CuO. (c) Charge density difference in DAT and *OH configurations. PDOS analysis of (d) DAT adsorption and (e) *OH adsorption on CuO and Cu sites of Pt@Cu/CuO configurations. (f) Adsorption energy calculation of DAT and *OH on various adsorption sites of Pt@Cu/CuO configurations. (g) Free energy calculation of DATOC intermediates on Cu/CuO and Pt@Cu/CuO. (h) Proposed DATOC reaction pathway on Pt@Cu/CuO.

Projected density of states (PDOS) was calculated to clarify how electronic interactions influence the adsorption characteristics between a catalyst and the intermediates during DATOC. A higher *d*‐band center (*ε*_d_) toward the Fermi level is indicative of strengthened adsorption of intermediates, due to the decreased filling of adsorbate‐metal antibonding states [61, 62]. As shown in Figure 5b, the *ε*_d_ of Cu NC and CuO sites in Pt@Cu/CuO shift upward to –1.693 and –2.055 eV, respectively, in comparison to those of Cu/CuO (–2.058 eV at Cu NC site and –2.285 eV at CuO site). This result suggests that the presence of Pt likely optimizes the adsorption/desorption energetics of the substrate or crucial reaction intermediates, thereby significantly accelerating DATOC performance [63]. Furthermore, the partial density of states were analyzed to reveal the interaction between the catalyst and adsorbates. As shown in Figure S20, a more extensive overlap with Cu 3d and O 2p orbitals near the Fermi level is observed in Pt@Cu/CuO compared to that of Cu/CuO, suggesting a pronounced substrates or intermediates adsorption capability on Pt@Cu/CuO due to the beneficial d–p coupling [64].

In addition, the charge distribution difference and Bader charge analysis between catalysts (Pt@Cu/CuO and Cu/CuO) and intermediates (DAT substrate and *OH species) were quantified analyzed (Figure 5c). The adsorption of DAT substrate and *OH on Pt@Cu/CuO induces charge redistribution on Cu NC and CuO sites to DAT (0.50 e^–^) and *OH (4.11 e^–^), respectively, which are superior to those of other sites on Pt@Cu/CuO and Cu/CuO (Figures S21and S22). The electron density difference result shows that the adsorption of *OH exhibits more electron transfer from the catalyst surface compared with DAT adsorption because of the low migration energy and small ionic radius [28]. This will induce the competitive adsorption of DAT and *OH to inevitably inhibit DAT activization. The Bader charge analysis indicates that the introduction of Pt NCs could induce multi‐sites of Pt/Cu/CuO heterostructure and balance co‐adsorption of DAT and *OH species.

Crystal orbital Hamiltonian population (COHP) was calculated to further evaluate the strength of chemical bonds [65]. The quantified M (Cu, Pt)–N (amino nitrogen in DAT) bond strength was calculated by integrated COHP (–ICOHP) up to the Fermi level. As shown in Figure 5d, the quantified –ICOHP values for Cu─N bonds in Cu NC and CuO are 1.14 and 1.12, while the –ICOHP value for Pt─N bond in Pt NC is 1.09. The higher –ICOHP value of Cu─N bond in Cu NC indicates a stronger DAT adsorption capacity on Cu NC site. In addition, the –ICOHP values of M (Cu, Pt)–O (oxygen in *OH) bonds are calculated as 1.99, 1.62, and 1.59 for Cu─O bond in CuO and Cu NC, and Pt─O bond in Pt NCs, respectively. The higher –ICOHP value of Cu─O bond of CuO site suggests a more inclined *OH adsorption on CuO site in Pt@Cu/CuO (Figure 5e).

To further verify the main active sites for DAT and *OH adsorption during DATOC, we calculated the adsorption energies of DAT and *OH on CuO, Cu NC, or Pt NC sites, respectively, in Pt@Cu/CuO and Cu/CuO (Figures 5f, S23, and S24). Both *OH and DAT starting substrates show negative adsorption energy values on each site of Pt@Cu/CuO and Cu/CuO. Typically, the adsorption energies of Cu NCs and CuO sites for DAT (–2.05 eV) and OH* (–5.44 eV) are the lowest, suggesting the most substantial adsorption of DAT and *OH on Cu NC and CuO sites in Pt@Cu/CuO, respectively. It may be due to the charge redistribution and electronic configuration in Pt/Cu/CuO interfaces constructed by electrochemical incorporation of Pt NC and electric field‐induced Cu/CuO heterostructures. Besides, the thermodynamic reaction mechanism of DATOC taking place on Pt@Cu/CuO and Cu/CuO is plotted and compared by free energy diagram (**Figures** 5g, S25, and S26). Based on the in situ ATR‐FTIR analysis, adsorption energy calculation, and electronic structure discussion, the possible *OH involvement and ─NH─NH─ oxidatively coupling pathway are discussed. It can be observed that the DAT dehydrogenation triggered by *OH could significantly reduce the free energy from 1.39 to –1.02 eV on Pt@Cu/CuO, confirming that the direct oxidation pathway, where high‐active *OH generated by OH^–^ adsorption promotes DAT dehydrogenation [27].

In addition, the rate‐determining step was determined as the dehydrogenation of ─NH─NH─ to form ─NH─N─ intermediate on both Pt@Cu/CuO and Cu/CuO. The introduction of Pt NC largely reduces the energy barrier from 1.63 to 0.53 eV, indicating that the kinetic of DATOC on Pt@Cu/CuO can be significantly promoted. The calculation analysis is consistent with the experimental results. The free energy calculations also reveal that the Pt NC site can stabilize N─N coupling intermediates by coordinating Pt/Cu NC with the triazole ring nitrogen, enabling fast dehydrogenation for DAAT formation. On the basis of the DFT calculations and operando characterizations, a plausible mechanism for the DATOC is illustrated in Figure 5h. The DAT and *OH are separately co‐adsorbed on Cu NC and CuO sites for electrochemical dehydrogenation of DAT triggered by active *OH. Then, the chemical N─N self‐coupling is followed by subsequent dehydrogenation leading to azo formation and DAAT product desorption. Pt NC plays a vital role (transmitter for charge transfer between catalysts and DAT substrates, and stabilizer for N─N coupling intermediates by coordinating Pt site with triazole nitrogen) in highly efficient DATOC.

### 2.5 Overall Electrolysis Performance

Given the thermodynamically favorable feature and accelerated kinetic of DATOC on Pt@Cu/CuO electrocatalyst, we further evaluate the performance in a two‐electrode H‐type electrolyzer. Pt@Cu/CuO and Pt/C are used as anode and cathode, respectively (Figure 6a). The assembled DATOC||HER system catalyzed by Pt@Cu/CuO||Pt/C achieves ultralow cell voltages of 0.876 and 0.959 V to reach 10 and 50 mA cm^−2^, respectively (Figure 6c). An apparent activity decay is observed by using CuO||Pt/C and CF||Pt/C electrodes, unequivocally demonstrating kinetically favorable feature of Pt@Cu/CuO for DATOC. After the reaction, the color of the anolyte changes to brownish red due to the formation of an azo chromophore group (Figure 6b).

**FIGURE 6:**
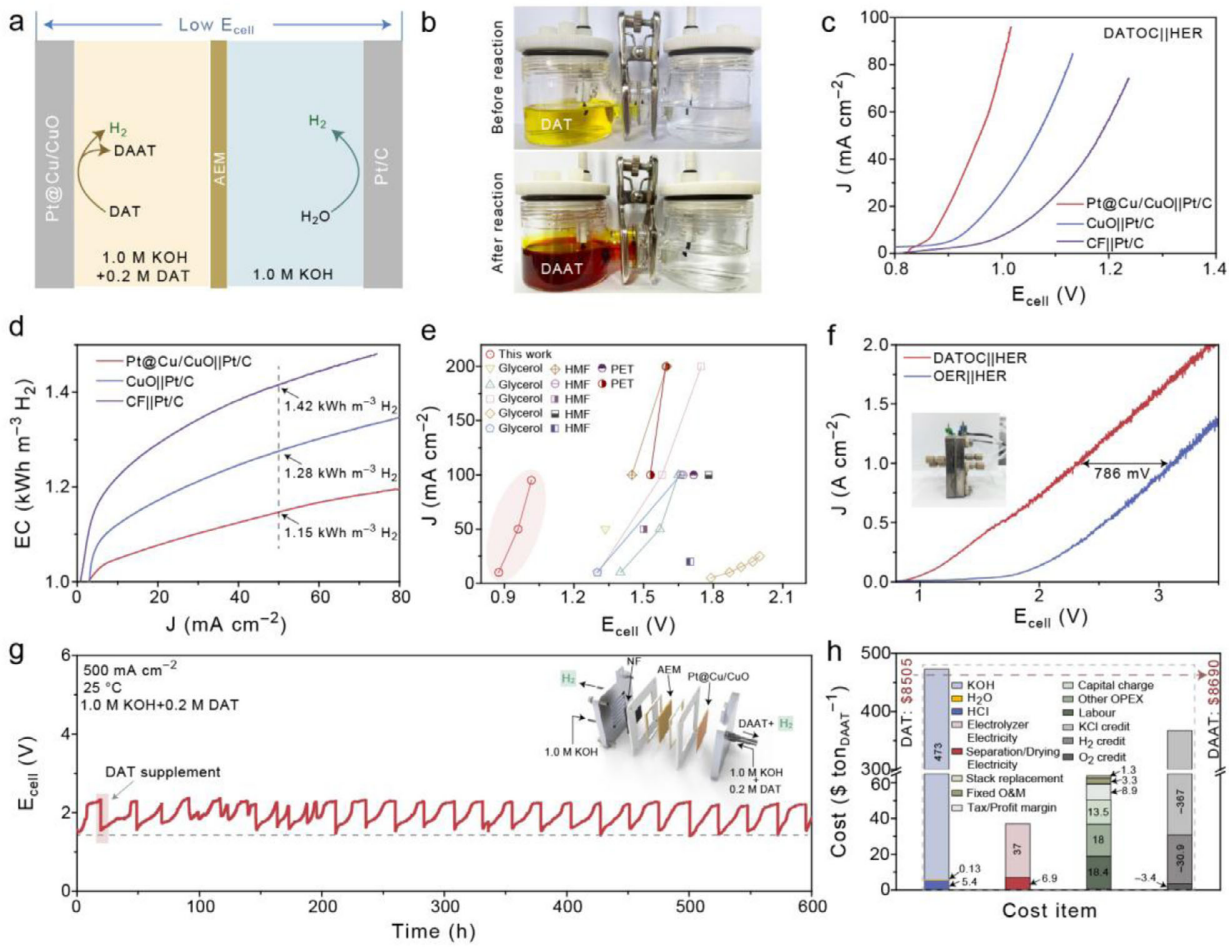
Evaluation for the performance of DATOC||HER dual‐electrode hydrogen production system. (a) Schematic illustration for the DATOC||HER coupled system. (b) Optical images of the H‐type electrolyzer before and after the reaction. (c, d) LSV curves and corresponding electricity consumption of DATOC||HER on Pt@Cu/CuO||Pt/C, CuO||Pt/C, and CF||Pt/C. (e) Comparison of the cell voltage at various current densities with the reported mainstream organic small molecules oxidation‐based water electrolysis. (f) LSV curves of DATOC||HER and OER||HER in a flow electrolyzer. (g) Long‐term stability of DATOC||HER at a current density of 500 mA cm^−2^ in 1.0 M KOH+0.2 M DAT. (h) Techno‐economic analysis of the DATOC||HER dual‐electrode hydrogen production system.

To further verify the thermodynamically favorable of DATOC, the energy consumption of each electrode for DATOC||HER coupling system is calculated (the detailed calculation method is shown in Supporting Information). The energy consumption of the DATOC||HER coupling system is calculated as only 1.15 kWh m^−3^ H_2_ at the current density of 50 mA cm^−2^, representing about 74% electricity input is saved compared with traditional water splitting (4.5–5.0 kWh m^−3^ H_2_)^[66]^ (Figure 6d). The significant reduction of electricity input is due to the one‐electron transfer process for one hydrogen molecule production. Such a low cell voltage and energy consumption are superior to those of state‐of‐the‐art mainstream organic small molecule (e.g., HMF, glycerol, PET, etc.) oxidations, indicating the thermodynamically and kinetically favorable feature of DATOC on Pt@Cu/CuO for value‐added azotriazole production (Figure 6e).

Leveraging this versatility, we further assemble an anion‐exchange membrane (AEM) flow electrolyzer using Pt@Cu/CuO and nickel foam as anode and cathode, respectively. The 1.0 M KOH and 1.0 M KOH containing 0.2 M DAT substrate are used as catholyte and anolyte, respectively (Figure 6f). The onset voltage of DATOC||HER electrolyzer needs only ∼0.85 V, which is about 650 mV lower than that of traditional OWS. In addition, the electrolyzer reduces the cell voltage by 786 mV compared with OWS to reach an industrial‐level current density of 1.0 A cm^−2^. Moreover, it can operate stably for at least 600 h at a high current density of 500 mA cm^−2^ without significant degradation, demonstrating the great potential for commercial deployment (Figure 6g). The post‐reaction structural characterizations of Pt@Cu/CuO and ICP‐OES analysis revealed relatively low levels of trace metal dissolution and surface reconstruction (Figure S27 and Table S3), while the overall framework of the catalyst was basically intact. The technical‐economic analysis (TEA) is then conducted to reveal the promising implementation of electrochemical coproduction of DAAT and hydrogen (**Figures** 6h **and** S28) [67]. Because there is no listed price of DAAT, we then evaluate the minimum selling price of DAAT from the cost of starting materials, electrolyte, electricity input for electrolysis, and product separation, as well as the revenues from produced H_2_ and recycle of electrolyte. It is noted that the DAT substrate (8505$ ton^−1^), electrolytes (478.53$ ton^−1^), and electricity input (43.9$ ton^−1^) account for the majority of total cost. By balancing the costs and revenues of each component, the cost of electrosynthesis of DAAT is calculated to 8690$ ton^−1^, which is close to that of DAT substrates. Based on the mechanism of DATOC, the production of DAAT molecules requires two stoichiometric DAT substrates, indicating that the cost of traditional synthesized DAAT is twice the price of DAT. More cost will be expended if including the chemical reagents and high‐temperature synthetic energy consumption. These results demonstrate that the current electrochemical coproduction of DAAT and hydrogen has potential for large‐scale operations.

## 3 Conclusion

In summary, we have successfully constructed Pt@Cu/CuO heterostructures for high selectivity and FE of DATOC. Benefiting from the low‐potential DATOC, the H* of dehydrogenation of DAT undergoes self‐coupling to form H_2_, promoting the electron and hydrogen atom economy. The proposed anodic H_2_ production from DATOC can balance the market mismatch of anode and cathode in traditional organic small molecule oxidations coupled low‐energy‐consumption H_2_ production system. Operando spectroscopy along with theoretical calculations reveal that the immobilization of Pt NCs in Cu/CuO mediates adsorption capacities for both *N‐*heterocyclic substrates/intermediates and OH^−^ species on Cu sites of Cu/CuO, collaboratively promoting DATOC kinetics. The synergistic adsorption of DAT and *OH on Cu NC and CuO sites promotes N─H activation of *NH_2_ in DAT, while the Pt NC regulates charge transfer between substrate and adsorbed molecules, and stabilizes the ─NH─NH─ intermediates via coordinating Pt/Cu with the N atom of tricyclazole. As a result, the energy barrier of DATOC is reduced on Pt@Cu/CuO heterostructures. Given this, the elaborate Pt@Cu/CuO exhibited as low as 0.832 V and 0.904 V_RHE_ to achieve 10 and 100 mA cm^−2^ with a high FE of 95% for DATOC. When coupled with HER, the assembled DATOC||HER system delivered ultralow cell voltages of 0.876 and 0.959 V to reach 10 and 50 mA cm^−2^, respectively. The corresponding energy input of the coupled system was reduced significantly by ∼74% compared to conventional water electrolysis. The practical application potential of DATOC||HER was evaluated by integrating flow electrolyzers and demonstrated outstanding durability for at least 600 h at a current density of 500 mA cm^−2^ without notable increase in applied cell voltage. This work elucidated the critical role of Pt‐mediated collaboratively adsorption of *OH and DAT on multiple Cu sites for highly efficient and long‐term stability of DATOC, providing guidance on a one‐stone, two‐birds strategy for dual‐electrode and energy‐saving hydrogen production, as well as green and eco‐friendly DAAT electrosynthesis.

## Author Contributions

**Jiachen Li**.: conceptualization, formal analysis, funding acquisition, writing – original draft, writing – review & editing; **Hanggu Su**., **Yuqiang Ma**., **Meng Li**., **Ruhao Zhang**., **Cihang Wang**., and **Zihang Zhao**.: formal analysis; **Jun Hu**.: software; **Xiaogang Mu**. and **Xuanjun Wang**.: writing – review & editing; **Haixia Ma**. and **Zhengxiao Guo**.: conceptualization, writing – review & editing, supervision, and funding acquisition.

## Conflicts of Interest

The authors declare no conflicts of interest.

## Supporting information




**Supporting File**: anie74400‐sup‐0001‐SuppMat.docx.

## Data Availability

The data that support the findings of this study are available from the corresponding author upon reasonable request.
