# Supplementary material for: Pt‐Mediated Co‐Adsorption of Hydroxyl and Nitrogen‐Heterocyclic Species Over Cu/CuO Heterostructure Enhances Coupled Azotriazole Electrosynthesis and Dual‐Electrode Hydrogen Production
Source: Angew Chem Int Ed Engl. 2026 Aug 23;65(42):e3766558. doi: 10.1002/anie.3766558 (PMC13653590; doi:10.1002/anie.3766558)
Supplement: Supplementary file 1 [file ANIE-65-e3766558-s001.docx]

**Pt-mediated Co-adsorption of Hydroxyl and Nitrogen-heterocyclic Species over Cu/CuO Heterostructure Enhances Coupled Azotriazole Electrosynthesis and Dual-electrode Hydrogen Production**

*Jiachen Li,* Hanggu Su, Yuqiang Ma, Meng Li, Ruhao Zhang, Cihang Wang, Zihang Zhao, Jun Hu, Xiaogang Mu, Xuanjun Wang, Haixia Ma,* Zhengxiao Guo,**

**Methods**

**Chemicals**

Nickel foam (*t*=300 μm) and copper foam (*t*=300 μm) were purchased from Suzhou ZhengTairong Electronic Materials Co., LTD. Potassium hydroxide (KOH, AR, 85%), Sodium hydroxide (NaOH, AR, ≥ 98%), Ammonium persulfate ((NH_4_)_2_S_2_O_8_, AR, ≥ 98%), chloroplatinic acid hydrate (H_2_PtCl_6_·6H_2_O, AR, Pt ≥ 37.5%), and 3,5-diamino-1,2,4-triazole (C_2_H_5_N_5_, ≥ 98%) were purchased from Shanghai Aladdin Biochemical Technology Co., Ltd.

**Preparation of the Pt@Cu/CuO electrocatalysts**

The Pt@Cu/CuO catalyst was synthesized via a two-step procedure. Prior to synthesis, commercial copper foam (CF, 1 × 0.5 cm^2^) was sequentially sonicated in acetone, 1.0 M HCl, and deionized water for 30 min to remove surface oxides. The pre-treated CF was subsequently rinsed with ethanol to improve surface wettability. The cleaned CF was immersed in an aqueous solution containing 3.0 M NaOH and 0.6 M (NH_4_)_2_S_2_O_8_ for 1 hour. Residual oxidants were removed by rinsing with distilled water and ethanol. The obtained CF-supported copper hydroxide nanowires (CF@Cu(OH)_2_) was then calcined at 300 °C for 4 h (ramp rate: 5 °C min^–1^) in a muffle furnace to obtain CuO@CF nanowires. Pt@Cu/CuO was subsequently prepared via electrodeposition strategy. A Pt plating solution (10 mg mL^–1^) was prepared using platinum (IV) chloride. Electrodeposition was performed in a three-electrode cell, the CF@CuO electrode, a carbon rod, and an Hg/HgO electrode served as the working, counter, and reference electrodes, respectively. The electrolyte consisted of 1.0 M KOH mixed with 2 mL of the Pt^4+^ solution. Electrodeposition of Pt was conducted via cyclic voltammetry (CV) between –0.924 and –1.424 V vs. Hg/HgO. The content of Pt in CuO@CF was controlled by adjusting the number of CV cycles. The resulting Pt@Cu/CuO catalyst was rinsed with deionized water and dried in vacuum oven. It was used directly as a self-supported anode for subsequent electrochemical DATOC testing.

**Preparation of the Pt@CC electrocatalysts**

The Pt@CC was prepared by one-pot electrodeposition method. Typically, 1.0 M KOH solution containing platinum (IV) chloride (10 mg mL^–1^) was used as electrolyte. Pretreated (washed by acetone, distilled water, and ethanol) carbon cloth (CC) is working electrode. The method of electrodeposition Pt is same as Pt@Cu/CuO as mentioned before.

**Material characterizations**

Scanning electron microscopy (SEM, Hitachi, SU8010) was performed to reveal the morphology of the catalysts. Transmission electron microscopy (TEM, FEI Talos F200X) and high-angle annular dark-field image-scanning transmission electron microscopy (HAADF-STEM) elemental mappings were performed to characterize microstructure of the catalysts. X-ray photoelectron spectroscopy (XPS, Thermo Scientific Nexsa) was carried out to determine the valance and electron transfer feature of the electrocatalysts. Inductively Coupled Plasma Optical Emission Spectrometer (ICP-OES, Agilent 5110) was conducted to identify the Pt and Cu element content in electrolyte and electrocatalysts. In situ Raman (Horiba, LabRAM HR Evol) was performed to elucidate the potential-dependent adsorption evolution of *N*-heterocyclic intermediates and hydroxyl species.

***In situ* differential electrochemical mass spectrometry (DEMS)**

*In situ* differential electrochemical mass spectrometry (DEMS, Shanghai Linglu Instruments) was employed to monitor the anodic gaseous products during the DATOC reaction. A polytetrafluoroethylene (PTFE) membrane was utilized to separate the liquid electrolyte from the high-vacuum system. This configuration blocked the liquid reagents and allowed the volatile species to permeate. The base pressure of the vacuum system was maintained below 1×10^-7^ Pa. Trace gaseous products generated from the DATOC process were continuously recorded.

***In situ* attenuated total reflection Fourier-transform infrared (ATR-FTIR)**

*In situ* attenuated total reflection Fourier-transform infrared (ATR-FTIR) spectroscopy was utilized to probe the reaction intermediates of the DAT-OCD process. Measurements were performed on a Nicolet IS50 spectrometer. The instrument was equipped with a Pike Technologies VeeMAX III accessory, a Jackfish J1 spectro-electrochemical cell, and a mercury-cadmium-telluride (MCT) detector. A gold (Au) film was deposited onto a silicon (Si) crystal via vacuum sputtering to serve as the conductive working support. To prepare the catalyst ink, the catalyst (collected from a 1×1 cm^2^ electrode) was dispersed in a solvent mixture. This mixture consisted of 0.5 mL of ethanol, 0.5 mL of deionized water, and 40 μL of a 5 wt% Nafion solution (D520). The suspension was sonicated to form a uniform ink. Finally, the ink was drop-cast onto the Au-coated Si support and dried in air prior to the measurements.

***Electrochemical characterization***

Electrochemical measurements were conducted using a CHI760e workstation (Shanghai Chenhua). Anodic DATOC was evaluated in a three-electrode configuration. Self-supported electrocatalysts, a graphite rod, and an Hg/HgO electrode were utilized as the working, counter, and reference electrodes, respectively. The electrolyte was 1.0 M KOH containing various concentrations of DAT (0 to 0.4 M). Linear sweep voltammetry (LSV) was performed from –0.2 to 0.8 V_Hg/HgO_ at a scan rate of 5 mV s^-1^. Chronopotentiometry (CP) was employed to assess catalyst stability. All measured potentials were converted to the reversible hydrogen electrode (RHE) scale using the equation:

*E(RHE) = E(Hg/HgO) + 0.098 V + 0.0591 × pH*

A two-electrode H-cell was assembled for concurrent DATOC and HER. Self-supported electrocatalysts and Pt/C@CC served as the anode and cathode, respectively. The anodic electrolyte was 1.0 M KOH containing 0.2 M DAT, and the cathodic electrolyte was 1.0 M KOH. The compartments were separated by a Fumasep FAA-3-50 anion-exchange membrane (AEM). Furthermore, an AEM flow cell was constructed using a 1.0 cm^2^ anode and cathode, separated by a AEM membrane. Nickel foam and Pt@Cu/CuO functioned directly as the cathodic and anodic gas diffusion electrodes, respectively. The cell was assembled without hot-pressing. Flow cell performance was evaluated at room temperature using a CHI760e workstation equipped with a current amplifier. During long-term chronopotentiometry at 500 mA cm^–2^, the cell voltage correlated with the consumption of DAT and water. Consequently, the electrolytes were periodically replenished. Voltage variations post-replenishment was recorded to evaluate operational stability. The product DAAT was isolated based on its pH-dependent solubility. The post-electrolysis mixture was stirred for 0.5 hour. Electrolyte was acidified to induce the precipitation of solid DAAT. The precipitate was directly collected via vacuum filtration. The obtained DAAT was sequentially washed with ice-cold water to remove residual acid. The isolated DAAT was dried at a low temperature for 24 hours. The dried solid was re-washed with ice-cold water until the filtrate reached a neutral pH. A subsequent drying step yielded the final DAAT powder.

***Electricity consumption calculation***

The electricity consumption (in kWh per m^3^ of H_2_) of the dual-electrode hydrogen production system for the DATOC||HER coupled system was calculated using the following equation:

$$W=\frac{n\times F\times U\times1000}{3600\times V_{m}}$$

Where *n* is the number of electrons transferred to produce each H_2_ molecule. Note that *n*=1 in our dual-electrode hydrogen production system, while *n*=2 for conventional water electrolysis. *U* is the applied cell voltage, and *V*_m_ (22.4 L mol^–1^) is the molar volume of gas under standard conditions. *F* is the Faraday constant (96485 C mol^–1^).

***Quantification of DAAT***

A calibration curve was established using the purified DAAT. 0.1 M DAAT stock solution was dispersed in 1.0 M KOH. Standard solutions with concentrations ranging from 0 to 0.25 mM were prepared via serial dilution. Absorbance was measured at 430 nm using a UV-Vis spectrophotometer (Lambda 750, PerkinElmer). A calibration curve (*R*^2^ > 0.999) was generated for product quantification.

Post-reaction electrolyte samples were diluted 50-fold with 1.0 M KOH to fit the linear range of the calibration curve. The absorbance at 430 nm was recorded, and DAAT concentrations were calculated based on the established calibration curve. The Faradaic efficiency (FE) for DAT oxidation were calculated using the following equations:

$$FE \left( \% \right)=\frac{n_{product}}{total passed charge/(4 \times F)}\times100\%$$

The content of DAT in electrolyte was quantitatively analyzed by ^1^H NMR. Deuterated DMSO was used as deuterated solvent. The concentration of the residual DAT in electrolyte was calculated based on the standard curve of DAT. The conversion, yield, and selectivity were obtained by the following equations:

$$Conversion \left( \% \right)=\frac{n_{initial substrate}-n_{residual substrate}}{n_{initial substrate}}\times100\%$$

$$Yield \left( \% \right)=\frac{n_{product}}{n_{initial substrate}}\times100\%$$

$$Selectivity \left( \% \right)=\frac{Yield (\%)}{Conversion (\%)}\times100\%$$

***Theoretical calculations***

Density functional theory (DFT) calculations were performed using the Vienna Ab initio Simulation Package (VASP). The generalized gradient approximation (GGA) with the Perdew-Burke-Ernzerhof (PBE) functional was employed to describe the exchange-correlation energy. A plane-wave cutoff energy of 500 eV was applied for all simulations. For the anode catalyst models, two specific configurations were constructed based on the CuO (111) facet: a CuO (111) slab supported with a Cu_6_ cluster (Cu_6_/CuO), and a CuO (111) slab co-supported with both Cu_6_ and Pt_6_ clusters (Pt_6_-Cu_6_/CuO). These models were utilized to investigate the DAT/*OH adsorption behavior and the DATOC reaction pathways. Structural optimizations were performed with full relaxation. The convergence criteria for energy and force were set to 10^–5^ eV and 0.05 eV Å^–1^, respectively. A vacuum layer of 15 Å was inserted along the z-axis to prevent artificial periodic interactions between slabs.

The Gibbs free energy (ΔG) was calculated using the following equation:

ΔG =ΔE_ads_+ΔE_ZPE_–TΔS

Here, ΔE_ads_, ΔE_ZPE_, and ΔS represent the adsorption energy, the change in zero-point energy, and the change in entropy, respectively. T is the standard temperature (298.15 K).

The adsorption energy (ΔE_ads_) was determined by the equation:

ΔE_ads_ = E_(*+ad)_–E_(*)_–E_(ad)_

In this equation, E_(*+ad)_, E_(*)_, and E_(ad)_ correspond to the total calculated energy of the adsorbed system, the bare catalyst surface, and the isolated adsorbate molecule, respectively.

**Figure S1.** Morphological characterizations of CF@Cu(OH)_2_ NWs. (a, b) SEM images. (c) High-resolution TEM, (d) HAADF-STEM and elemental mappings.

**Figure S2.** SEM images of (a) CuO@CF and (b) Pt@Cu/CuO@CF.

**Figure S3.** XRD patterns of (a) Cu(OH)_2_@CF and (b) CuO@CF, Cu/CuO@CF, and Pt@Cu/CuO@CF.

**Figure S4.** Morphological characterizations of Cu/CuO@CF. (a, b) SEM images. (c, d) TEM and HRTEM images. (e) HAADF-STEM image and elemental mappings. (f) EDS spectrum.

**Figure S5.** LSV curves of DATOC on Pt@Cu/CuO under various DAT concentration.

**Figure S6.** Optimization of (a, b) Pt ion concentration and (c, d) the number of CV cycles for Pt NCs deposition toward DATOC activity.

**Figure S7.** Morphological characterizations of CC@Pt NCs. (a, b) SEM images. (c, d) TEM and HRTEM images.

**Figure S8.** (a) UV-Vis spectrophotometry at various concentrations of DAAT. (b) The fitted line of absorbance versus concentration of DAAT.

**Figure S9.** UV-Vis spectrophotometry of electrolyte during DATOC on Pt@Cu/CuO. (a) Applied potentials versus wavelength and (b) electrolysis time versus wavelength at 0.9 V_RHE_.

**Figure S10.** UV-Vis spectrophotometry of the electrolyte during DATOC catalyzed by (a) Cu/CuO and (b) CuO over the applied potentials of 0.9–1.4 V.

**Figure S11**. (a) ^1^H NMR of DAT with different concentrations. (b) Corresponding linear fitting for different concentrations of DAT. (c) Conversion rate of DAT, selectivity, and yield of DAAT at various applied potentials.

**Figure S12.** Structural characterizations of Pt@Cu/CuO after 2000 CV cycles. (a) SEM. (b, c) TEM and HRTEM images. (d) HAADF-STEM and elemental mappings of Cu, Pt, O. High-resolution XPS spectra of (e) Cu 2p and (f) Pt 4f signals.

**Figure S13.** Stability test of DATOC||HER in H-type electrolyzer.

**Figure S14.** DEMS spectra of DATOC on Cu/CuO.

**Figure S15.** DEMS spectra of cathodic HER.

**Figure S16.** In situ FTIR spectra of DATOC on Cu/CuO.

**Figure S17.** In situ FTIR spectra of DATOC on Pt@Cu/CuO and Cu/CuO.

**Figure S18.** (a) Variations of OCP of Pt@Cu/CuO and CuO in 1.0 M KOH with the injection of 0.2 M DAT. (b) CV curves of Pt@Cu/CuO and CuO in 1.0 M KOH+0.2 M DAT.

**Figure S19.** (a) OCP, (b) CV curves, and (c) Zeta potential measurements of different amount of Pt loading on Cu/CuO.

**Figure S20.** PDOS of Cu 3d, O 2p in Pt@Cu/CuO and Cu/CuO.

**Figure S21.** The differential charge density models of DAT and *OH adsorbed on Pt and Cu NCs sites of Pt@Cu/CuO.

**Figure S22.** The differential charge density models of DAT and *OH adsorbed on CuO and Cu NCs sites of Cu/CuO.

**Figure S23.** The structural models of DAT and *OH adsorbed on Cu NCs, CuO, and Pt sites of Pt@Cu/CuO.

**Figure S24.** The structural models of DAT and *OH adsorbed on Cu NCs and CuO sites of Cu/CuO.

**Figure S25.** Structural model of *OH and DAT substrate co-adsorption on Cu/CuO.

**Figure S26.** Structural models of DATOC intermediates adsorbed on Cu/CuO.

**Figure S27.** Morphological and structural characterizations of Pt@Cu/CuO after long-term stability tests in flow electrolyzer. (a) SEM, (b) TEM, (c) HRTEM, (d) HAADF-STEM and elemental mapping. High-resolution XPS spectra of (e) Cu 2p, (f) Cu LMM, and (g) Pt 4f signal.

SEM image of the stability-tested Pt@Cu/CuO catalyst, anode shows original Cu/CuO nanowire structures with some aggregation of the nanowires and small cracks; the latter may be due to the erosion effect during the long-term stability testing (Figure S27a). TEM image further confirms the retention of nanowire along with the immobilized Pt NCs. HRTEM images display the lattice distance of 0.186 nm, 0.21 nm, and 0.221 nm, attributable to CuO (–202), CuO (200), and Pt (111) planes, respectively (Figure S27b, c). HAADF-STEM and elemental mapping reveal the uniform distribution of Cu, O, and Pt elements along the nanowire (Figure S27d). High-resolution XPS spectra of Cu 2p signal exhibits a sharp peak at 931.8 eV, corresponding to the Cu 2p_3/2_ signal for Cu^+^/Cu^0^ species.^[1]^ Other peaks located at 934.0 eV and 942.1/944.3 eV are ascribed to Cu 2p_3/2_ signal for Cu^2+^ and fingerprint identification of Cu^2+^ satellite peaks (Figure S27e).^[2]^ Due to the difficulty in distinguishing Cu^+^/Cu^0^ species from Cu 2p spectra, we further conducted Cu LMM auger spectra of Pt@Cu/CuO after long-term stability test (Figure S27f). The peaks located at 915.8 eV and 917.0 eV are attributed to the Cu^+^ and Cu^2+^, respectively.^[3]^ Moreover, the doublets in high-resolution XPS spectrum at 71.2 eV and 73.2 eV are assigned to the Pt 4f_7/2_ and Pt 4f_5/2_ of Pt^0^ and surface oxidized Pt^𝛿+^ (Figure S27g).^[4]^ ICP-OES was performed to detect the possible dissolve metal ions in electrolyte. ICP-OES measurements of the electrolyte after long-term stability test revealed partial catalyst dissolution. Based on the above discussion, although surface reconstruction and metal leaching occurred during long-term stability test, the overall framework of the catalysts was basically intact.

**Figure S28.** Mass flow of the recycling process.

**Table S1.** Comparison of potential at 10 mA cm^–2^ for DATOR with recently reported anodic chemicals upgradation reactions.

| Substrate | Electrode materials | Potential at 10 mA cm^–2^ (V vs. RHE) | Reference |
| --- | --- | --- | --- |
| **DAT** | **Pt@Cu/CuO** | **0.832** | ***This Work*** |
| Glycerol | PMo_12_@MOF-74 | ~1.1 | *Adv. Funct. Mater.* **2025***, 2502616* |
| Glycerol | NiCoFeRuRe | 1.133 | *Angew. Chem. Int. Ed.* **2025***, e202501766* |
| Glycerol | Cu-NiCo/NF | 1.23 | *Angew. Chem. Int. Ed.* **2024***, e202411542* |
| 5-amino-1*H*-tetrazole (5-AT) | NiO/CC-500 | 1.23 | *Inorg. Chem.* **2025***, 64, 4544–4554* |
| Benzyl alcohol (BA) | Co-Ni_2_P/NF | ~1.26 | *Adv. Funct. Mater.* **2025***, 2510160* |
| Butane-1,4-diol (BD) | Mo_1_-CoSe/NF | ~1.28 | *Adv. Mater.* **2025***, 37, 2419058* |
| Ethylene glycol (EG) | NiFe-sc-PBA | ~1.30 | *Nat. Commun.* **2025***, 16, 3458* |
| HMF | E-CuO | ~1.30 | *ACS Catal.* **2025***, 15, 5968−5981* |
| Glucose | Al(OH)_3_/Co(OH)_2_ | ~1.30 | *Adv. Energy Mater.* **2024***, 14, 2402789* |
| Glycerol | Rh_1_-In_2_O_3_ | ~1.30 | *Small* **2025***, 2500123* |
| Benzyl alcohol (BA) | Ni_0.67_V_0.33_-LDH/ACT | ~1.30 | *Nat. Commun.* **2025***, 16, 266* |
| Propylamine | (Ni_x_Co_1−x_)Se_2_-R | 1.31 | *J. Am. Chem. Soc.* **2024***, 146, 27090−27099* |
| Ethylene glycol | NF/Ni(OH)_2_-adp | 1.32 | *Green Chem.,* **2025***, 27, 6512–6524* |
| HMF | Pt/CuO@CF | ~1.33 | *Adv. Mater.* **2025***, 2417684* |
| HMF | NiCuO_x_/NF | ~1.33 | *ACS Energy Lett.* **2025***, 10, 571−578* |
| HMF | CoO_x_ | ~1.34 | *Adv. Funct. Mater.* **2025***, 2502081* |
| HMF | Mo-doped NiFeB | 1.35 | *Angew. Chem. Int. Ed.* **2025***, e202424345* |
| Methanol | F_10_-Ni_3_N | ~1.35 | *Adv. Mater.* **2025***, 2507573* |
| Benzylamine | Fe-Ni_3_S_2_ | 1.36 | *Adv. Funct. Mater.* **2023***, 2301884* |
| Glycerol | Ni_3_(HHTQ)_2_ | 1.36 | *Angew. Chem. Int. Ed.* **2025***, e202502425* |
| Butylamine | CoSe_2_−S_V_ | 1.37 | *ACS Catal.* **2022***, 12, 11391−11401* |
| Methanol | Ni_3_FeN-Ru_buried_ | ~1.37 | *Nat. Commun.* **2025***, 16, 286* |
| Pyrazole | Ni-BDC | 1.37 | *Angew. Chem. Int. Ed.* **2024***, e202402176* |
| HMF | NiMoCe/NF | ~1.38 | *Angew. Chem. Int. Ed.* **2025***, e202506017* |
| HMF | CoNiMnMoPd | ~1.38 | *Angew. Chem. Int. Ed.* **2025***, e202502776* |
| Cyclohexenylethylamine (CHE) | NiO/NC | 1.38 | *Adv. Energy Mater.* **2024***, 2400374* |
| HMF | NiO | ~1.40 | *ACS Catal.* **2025***, 15, 1727−1738* |
| Methanol | Cr_0.02_Ni(OH)_2+δ_ | ~1.40 | *ACS Catal.* **2024***, 14, 16234−16244* |
| Glycerol | Cu_3_N-Pd | 1.51 | *Angew. Chem. Int. Ed.* **2025***, e202425006* |
| Benzylamine | Vo-CuO/CF | ~1.52 | *Chem. Sci.,* **2024***, 15, 12580–12588* |

**Table S2.** Comparison of cell voltage at 10 mA cm^–2^ for DATOR||HER with recently reported chemicals upgradation coupled water electrolysis.

| Substrate | Electrode materials | Current density (mA cm^–2^) | Cell voltage (V) | Reference |
| --- | --- | --- | --- | --- |
| **0.2 M DAT** | **Pt@Cu/CuO\|\|Pt/C** | **10** | **0.876** | ***This Work*** |
|  |  | **50** | **0.959** |  |
|  |  | **95** | **1.016** |  |
| 0.1 M Glycerol | Ni_3_S_2_/Co_9_S_8_/FeNiN\|\|NiMoN | 50 | 1.335 | *Energy Environ. Sci., 2025,18, 7695-7707* |
| 0.1 M Glycerol | Cu-NiCo/NF\|\|Cu-NiCo/NF | 10 | 1.40 | *Angew. Chem. Int. Ed. 2024, e202411542* |
|  |  | 50 | 1.57 |  |
|  |  | 100 | 1.65 |  |
| 0.1 M Glycerol | NiCoFeRuRe\|\|NiCoFeRuRe | 10 | 1.304 | *Angew. Chem. Int. Ed. 2025, e202501766* |
|  |  | 100 | 1.579 |  |
|  |  | 200 | 1.748 |  |
| 0.1 M Glycerol | Ni_3_(HHTQ)_2_\|\|Ni_3_(HHTQ)_2_ | 5 | 1.79 | *Angew. Chem. Int. Ed. 2025, e202502425* |
|  |  | 10 | 1.87 |  |
|  |  | 15 | 1.92 |  |
|  |  | 20 | 1.97 |  |
|  |  | 25 | 2 |  |
| 0.1 M Glycerol | PMo_12_@MOF-74\|\|MoC@CoO | 10 | 1.30 | *Adv. Funct. Mater. 2025, 2502616* |
|  |  | 100 | 1.66 |  |
| 0.1 M HMF | NiMoCe/NF\|\|NiPt_4_/NM | 100 | 1.45 | *Angew. Chem. Int. Ed. 2025, e202506017* |
|  |  | 200 | 1.60 |  |
| 0.1 M HMF | CuO@ZIF-Co(g)\|\|CuO@ZIF-Co(g) | 100 | 1.67 | *Adv. Energy Mater. 2025, e04886* |
| 10 mM HMF | F-V_O_-CoMoO_4_\|\|Pt/C | 50 | 1.50 | *Angew. Chem. Int. Ed. 2025, e202513396* |
| 50 mM HMF | Mn-Ni(OH)_2_/PMS | 100 | ~1.78 | *Angew. Chem. Int. Ed. 2025, e202511868* |
| 0.1 M HMF | NiV LDH/NF\|\|NiMo/NF | 20 | ~1.7 | *ACS Catal. 2025, 15, 14693−14702* |
| 0.1 M PET | NF/CoP\|\|NF/Ni(OH)_2_-adp | 100 | 1.715 | *Green Chem., 2025, 27, 6512–6524* |
| 0.1 M PET | Mo_1_-CoSe-VMo/NF | 100 | ~1.53 | *Adv. Mater. 2025, 37, 2419058* |
|  |  | 200 | 1.596 |  |

**Table S3.** Elemental leaching analysis from ICP-OES after long-term (600 hours) flow cell stability test of DATOC||HER.

| Entry | Object | Content | |
| --- | --- | --- | --- |
|  |  | Cu | Pt |
| 1 | CF@Cu/CuO@Pt (mg) | 23.88542153 | 1.374876 |
| 2 | Solution after prolonged reaction (mg) | 0.461638218 | 0.001256 |
| 3 | Leaching rate (%) after 600 hours | 1.932719577 | 0.091386 |

***Techno-economic analysis (TEA)***

In this study, it was assumed that the electrochemical device consumed 50 MW of power to produce hydrogen and DAAT through water electrolysis. The annual operating time is 330 days, and the stack operates under constant temperature and pressure conditions with the electrolyte solution concentration maintained at 1.0 M KOH. During the process, the Faraday efficiency and the hydrogen generation rate remained constant. Batteries and related equipment operate at a constant capacity and provide continuous power during the operational period.

This analysis determines the minimum selling price (MSP) of the DAAT by quantifying the capital and operational costs of the electrolysis unit at a total power output of 50 MW. MSP is defined as the price at which the net present value (NPV) becomes zero under a 20-year period with an internal rate of return (IRR) of 10%. Through sensitivity analysis and scenario analysis, the effects of factors such as current density, capital cost, separation cost, and product price were investigated.

**Table S4** Baseline operating conditions used for technoeconomic analysis (TEA) of DAAT production.

| **Operating conditions** | **Value** |
| --- | --- |
| Voltage (V) | 1.6 |
| H_2_ selectivity (%) | 100 |
| DAAT selectivity (%) | 60 |
| DAT conversion (%) | 0.6 |
| Anolyte (KOH) concentration (M) | 1.0 |
| Catholyte (KOH) concentration (M) | 1.0 |
| Electrons needed per mole of DAAT (n) | 4 |

**Table S5** Factory Scale Parameters

| **Components** | **Values** |
| --- | --- |
| Factory scale (MW) | 50 |
| Annual operating days (d) | 330^[5,6]^ |
| Total Operating Current (A) | 31,250,000 |
| Total Operating Charge (C) | 8.91E+14 |
| Total number of electronic operations (mol) | 9234596051 |
| Number of electrons used in the synthesis of DAAT (mol) | 5540757631 |
| Annual DAAT production moles (mol/year) | 1385189408 |
| Total annual DAAT production quality (t/year) | 268726.7451 |

**Table S6** Mass balance calculated based on parameters summary.

| **Components** | **Values (t/y)** |
| --- | --- |
| H_2_ production | 5540.757631 |
| O_2_ production | 0.46639374 |
| water required | 49866.81868 |
| DAT required | 304741.6697 |
| KOH required | 155141.2137 |
| HCl required | 98348.44795 |
| KCl production | 206531.7407 |

**Table. S7** Capital expenditure decomposition.

| **Type** | **Comments** | **Values (dollar)** |
| --- | --- | --- |
| Electrolyzer system | Stack cost | 22500000^[7-9]^ |
|  | Balance of plant (BOP) | 22500000^[7-9]^ |
|  | Installation cost | 5400000 |
| Total fixed capital investment | Indirect costs | 6646425.754 |
|  | Contingency costs | 6646425.754 |
|  | Direct costs | 66464257.54 |
|  | Residual value | 7975710.905 |
| Downstream separation and storage（Aspen Process Economic Analyzer V10.0.） | Flash tank | 451817.5583 |
|  | Neutralization reactor | 5182613.169 |
|  | Crystallization pond | 3860168.038 |
|  | Solid-liquid separation | 1518775.72 |
|  | dryer | 1616941.015 |
|  | H_2_ storage tank | 1137928.669 |
|  | O_2_ storage tank | 1399665.638 |
|  | Heat exchange network | 896347.7366 |

**Table S8** Annual Operating Expenditure (OPEX) Breakdown

| Raw materials | DAT cost (dollar) | 2285673924 |
| --- | --- | --- |
|  | KOH cost (dollar) | 127215795.2 |
|  | HCl cost (dollar) | 1448279.244 |
|  | High‑purity water cost (dollar) | 34906.77307 |
| Energy | Electrolyzer electricity cost (dollar) | 19800000 |
|  | Separation & drying electricity cost (dollar) | 391000 |
|  | Drying gas cost (dollar) | 66000000 |
| Fixed costs | Fixed O&M (3% of direct cost, dollar) | 1993927.726 |
|  | Labor cost (50 people) | 9900000 |
|  | Stack replacement (15% of CAPEX, 5 years, dollar) | 675000 |
|  | Other operating costs (15% of FCI, dollar) | 11963566.36 |

**Notes:** Air flow rate: 100 t/h, Compressed air energy consumption: 0.1 kWh/N m^3^, Air density: 1.2 g/cm^3^

**Table S9** Income Schedule

| Sources of Income | Value(dollar) | |
| --- | --- | --- |
| Main product DAAT unit price | | *P* |
| DAAT annual revenue | | 268726.7451**P* |
| KCl annual revenue | | 98616840.86 |
| H_2_ annual revenue | | 8311136.446 |
| O_2_ annual revenue | | 229017.9821 |

**Table S10** Key material, energy flow rates, and prices for an electrochemical plant with the capacity of DATOC||HER

| Materials or Energy | Price ($/t or $/KWh) | | Ref. |
| --- | --- | --- | --- |
| KOH | | 830 | ^[10]^ |
| H_2_ | | 1500 | ^[11, 12]^ |
| KCl | | 477.49 | ^[13]^ |
| Process Water | | 0.2 | ^[14]^ |
| Cooling water | | 1.39 | ^[14]^ |
| High purity H2O ($/t) | | 0.7 | ^[15]^ |
| DAT | | 820 | ^[13]^ |
| HCl | | 14.726 | ^[13]^ |
| Steam price | | 4.22 | ^[16]^ |
| Electricity price | | 0.05 | ^[5]^ |
| O_2_ | | 50 | ^[13]^ |

**Calculation details**

**Calculate the total current:**

$I=\frac{P}{U}=\frac{50\times{10}^{6}}{1.6}=31250000$ (A)

**Calculate the total annual charge (ignoring efficiency):**

$t=330\times24\times3600=28512000$ (s)

$Q=I\times t=31250000\times28512000=8.91\times{10}^{14}$ (C)

**Calculate the molar amount of electrons (Faraday's constant F=96485 C/mol)**

$n_{e^{-}}=\frac{Q}{F}=\frac{8.91\times{10}^{14}}{96485}=9.23\times{10}^{9}$ (mol e^–^)

**Calculate the molar amount of DAAT (4 electrons required per molecule)**

$n_{\mathrm{DAAT}}=\frac{n_{e^{-}}}{4}*0.6=\frac{9.23\times{10}^{9}}{4}=1.39\times{10}^{9}$ (mol)

**Calculate the mass of DAAT (M=**$\boldsymbol{194\times}\boldsymbol{10}^{\boldsymbol{-6}}$ **t/mol)**

$m_{\mathrm{DAAT}}=n_{\mathrm{DAAT}}\times M_{\mathrm{DAAT}}=2.31\times{10}^{9}\times194\times{10}^{-6}=2.69\times{10}^{5}$ (t)

All subsequent material balance, energy balance, and economic balance calculations are based on the total annual production volume of DAAT, When the selling price of DAAT is *P*, all expenditures and all revenues in this calculation process are equal, resulting in zero net profit (NPV = 0), from which the MSP of DAAT is derived.

To calculate the cost components, the following equations are used:

**Stack Cost =Balance of Plant (BOP) cost**:

Unit Price of Electricity per kW × Scale of the Factory

**Total Cost of Electrolyzer (Uninstalled):**

Stack Cost + BOP cost

**Installation costs**:

12% × Total Cost of Electrolyzer^[9]^

**Total Cost of Electrolyzer (Installation):**

1.2×Total Cost of Electrolyzer (Uninstalled)

**Direct Costs**:

Downstream Separation Equipment Costs + Total Cost of Electrolyzer (Installation)

**Indirect costs** ^[17]^ **= Contingency costs**:

10 % × Direct Costs ^[18]^

**Fixed Asset Investment**:

Direct Costs + Indirect costs + Contingency costs

**Fixed Operation and Maintenance Costs:**

Total Cost of Electrolyzer (Uninstalled) × 3%^[5,6,8]^

**Stack annual replacement cos**t:

$\frac{Initial Stack Cost\times Replacement Factor}{Replacement Cycle}$^[19]^

**References**

[1] G Ertl, R Hierl, H Knözinger, N Thiele, HP Urbach, XPS study of copper aluminate catalysts, *Appl. Surf. Sci*., 1980, 5, 49–64.

[2] R. P. Vasquez, CuO by XPS, *Surf. Sci. Spectra*, 1998, 5, 262.

[3] Zhang, W., Huang, C., Xiao, Q., Yu, L., Shuai, L., An, P., Zhang, J., Qiu, M., Ren, Z., Yu, Y., Atypical Oxygen-Bearing Copper Boosts Ethylene Selectivity toward Electrocatalytic CO_2_ Reduction, *J. Am. Chem. Soc*. 2020, 142, 11417−11427.

[4] Li, J., Qiu, R., Zhang, S., Peng, L., Dong, Y., Jiang, Y., Li, Y., Fang, N., Yu, J., Dong, J., Zheng, H., Ding, L., Wan, J., Akpinar, I., Kuang, J., Chen, G., Ye, J., Sun, Y., Lin, L., Zheng, S., Yang, S., Li, J., Li, J.F., Synergistically Enhanced Co-Adsorption of Reactant and Hydroxyl on Platinum-Modified Copper Oxide for High-Performance HMF Oxidation, *Adv. Mater*. 2025, 2417684.

[5]	Jouny, M., Luc, W., Jiao, F., General Techno-Economic Analysis of CO_2_ Electrolysis Systems. *Ind. Eng. Chem. Res*., 2018, 57, 2165–2177.

[6]	Kuckshinrichs, W., Ketelaer, T., Koj, J. C. Economic analysis of improved alkaline water electrolysis. *Front. Energy Res.*, 2017, 5, 1.

[7]	Bourne, S. The future of fuel: The future of hydrogen. *Fuel Cells Bull.*, 2012, 12–15.

[8]	Buttler, A., Spliethoff, H. Current status of water electrolysis for energy storage, grid balancing and sector coupling via power-to-gas and power-to-liquids: A review. *Renew. Sust. Energy Rev.*, 2018, 82, 2440–2454.

[9]	Ruth, M., Mayyas, A., Mann, M. Manufacturing Competitiveness Analysis for PEM and Alkaline Water Electrolysis Systems. National Renewable Energy Laboratory (NREL), Golden, CO (United States), 2019.

[10]	Zech, K. M., Dietrich, S., Reichmuth, M., Weindorf, W., Müller-Langer, F. Techno-economic assessment of a renewable bio-jet-fuel production using power-to-gas. Appl. Energy, 2018, 231, 997–1006.

[11]	Parkinson, B., Balcombe, P., Speirs, J. F., Hawkes, A. D., Hellgardt, K. Levelized cost of CO_2_ mitigation from hydrogen production routes. Energy Environ. Sci., 2019, 12, 19–40.

[12]	Glenk, G., Reichelstein, S., Economics of converting renewable power to hydrogen. Nat. Energy, 2019, 4, 216–222.

[13]	100ppi.com. (n.d.).. Retrieved from https://www.100ppi.com/mprice/plist-1-819-1.html

[14]	M. R. Rover, A. Aui, M. M. Wright, R. G. Smith and R. C. Brown, Green Chem., 2019, 21, 5980-5989.

[15]	M. A. Khan, T. Al-Attas, S. Roy, M. M. Rahman, N. Ghaffour, V. Thangadurai, S. Larter, J. Hu, P. M. Ajayan, M. G. Kibria. Seawater electrolysis for hydrogen production: a solution looking for a problem? Energy Environ. Sci., 2021, 14, 4831–4839.

[16] Steward, D., Ramsden, T., Zuboy, J. H_2_A Central Hydrogen Production ModelVersion 3 User Guide. Natl. Renew. Energy Lab. 2012.

[17]	P. H. Kobos, T. E. Drennen, A. V. Outkin, E. K. Webb, S. M. Paap, S. Wiryadinata. Techno-Economic Analysis: Best Practices and Assessment Tools. Sandia Report. 2020.

[18] Turton, R., Bailie, R. C., Whiting, W. B., & Shaeiwitz, J. A. (2008). Analysis, synthesis and design of chemical processes. Pearson education.

[19]	Report, F. Analysis of polystyrene stars by GPC/viscometry. LC-GC North America, 2005, 23, 68–69.
